# Distinct DNA Binding Sites Contribute to the TCF Transcriptional Switch in *C. elegans* and *Drosophila*

**DOI:** 10.1371/journal.pgen.1004133

**Published:** 2014-02-06

**Authors:** Chandan Bhambhani, Aditi J. Ravindranath, Remco A. Mentink, Mikyung V. Chang, Marco C. Betist, Yaxuan X. Yang, Sandhya P. Koushika, Hendrik C. Korswagen, Ken M. Cadigan

**Affiliations:** 1 Department of Molecular, Cellular and Developmental Biology, University of Michigan, Ann Arbor, Michigan, United States of America; 2 Hubrecht Institute, Royal Netherlands Academy of Arts and Sciences and University Medical Center Utrecht, Utrecht, The Netherlands; 3 Department of Biological Sciences, Tata Institute of Fundamental Research, Colaba, Mumbai, India; Harvard University, United States of America

## Abstract

Regulation of gene expression by signaling pathways often occurs through a transcriptional switch, where the transcription factor responsible for signal-dependent gene activation represses the same targets in the absence of signaling. T-cell factors (TCFs) are transcription factors in the Wnt/ß-catenin pathway, which control numerous cell fate specification events in metazoans. The TCF transcriptional switch is mediated by many co-regulators that contribute to repression or activation of Wnt target genes. It is typically assumed that DNA recognition by TCFs is important for target gene location, but plays no role in the actual switch. TCF/Pangolin (the fly TCF) and some vertebrate TCF isoforms bind DNA through two distinct domains, a High Mobility Group (HMG) domain and a C-clamp, which recognize DNA motifs known as HMG and Helper sites, respectively. Here, we demonstrate that POP-1 (the *C. elegans* TCF) also activates target genes through HMG and Helper site interactions. Helper sites enhanced the ability of a synthetic enhancer to detect Wnt/ß-catenin signaling in several tissues and revealed an unsuspected role for POP-1 in regulating the *C. elegans* defecation cycle. Searching for HMG-Helper site clusters allowed the identification of a new POP-1 target gene active in the head muscles and gut. While Helper sites and the C-clamp are essential for activation of worm and fly Wnt targets, they are dispensable for TCF-dependent repression of targets in the absence of Wnt signaling. These data suggest that a fundamental change in TCF-DNA binding contributes to the transcriptional switch that occurs upon Wnt stimulation.

## Author Summary

The DNA of cells must be correctly “read” so that the proper genes are expressed. Transcription factors are the primary “DNA readers”, and these proteins bind to specific DNA sequences. Using nematodes as a model system, we investigated the rules of DNA binding for a particular transcription factor, called POP-1, which mediates Wnt signaling, an important cell-cell communication pathway. In addition to its known DNA binding site, we found that POP-1 recognizes additional sequences, termed Helper sites, which are essential for activation of Wnt targets. We used this knowledge to discover that Wnt signaling is active in pacemaker cells in the nematode intestine, which control defecation, a rhythmic behavior with parallels to the vertebrate heartbeat. POP-1 has a dual role in regulating Wnt targets, repressing target genes in the absence of signaling and activating them upon signal stimulation. Surprisingly, we found that Helper sites are only required for activation and not repression, and that this is also the case in the fruit fly *Drosophila*. This work thus reveals an unexpected complexity in POP-1 DNA binding, which is likely to be relevant for its human counterparts, which play important roles in stem cell biology and cancer.

## Introduction

Transcriptional switches are common in the regulation of gene expression by cell-cell signaling pathways [1]. This mechanism is typified by active repression of transcription under basal conditions, which is converted to activation by the respective signaling pathway. Examples include the Notch and Wnt/ß-catenin signaling pathways [1], [2], as well as class II nuclear hormone receptors [3]. These switches are important to ensure the proper pattern of expression in development [4], [5] and physiology [6].

The T-cell factor (TCF) family of transcription factors (TFs) offers a prominent example of a transcriptional switch [7]. TCFs are major nuclear mediators of Wnt/ß-catenin signaling, which controls numerous cell fate decisions during development and whose misregulation has been linked to cancer and other human pathologies [8]–[11]. Wnt signaling promotes the stabilization and nuclear accumulation of β-catenin [12], [13]. In the nucleus, ß-catenin is recruited to Wnt response elements (WREs), the *cis*-regulatory modules that control Wnt target gene transcription, through direct binding to TCFs [7], [14]. TCFs recognize DNA through their High Mobility Group (HMG) domain and act as repressors of gene transcription in the absence of ß-catenin. However, when bound by ß-catenin, they become transcriptional activators [7], [9], [14].

*Drosophila* and *C. elegans* each possess a single *TCF* gene, *TCF/Pangolin* (*TCF/Pan*) in flies and *pop-1* in nematodes. Genetic evidence indicates that both these TCFs operate as transcriptional switches. For example, in *C. elegans* embryos, Wnt signaling activates transcription of *end-1* and other endoderm-specific genes in the E blastomere [15]–[18]. The adjacent MS blastomere, which does not receive Wnt signal, doesn't express endoderm genes and develops into mesoderm [15]–[20]. In *pop-1* mutants, there is a reduction of *end-1* expression in E cells [16], [18] while MS cells now express *end-1* and other endoderm markers [15]–[18], [20], leading to both blastomeres adopting an endoderm-like fate [19]–[23]. Similarly, mutation of a single HMG binding site in a *end-1* WRE reporter results in a similar pattern of GFP expression as seen in *POP-1* mutants [16]. Similar data are found in flies, where loss of *TCF/Pan* results in both loss of activation and expansion of Wnt targets [24], [25] and mutation of HMG sites in WRE reporters display loss of activation as well as derepression of expression [26]–[28]. Thus POP-1 and TCF/Pan repress Wnt targets in the absence of signaling and activate the same genes upon Wnt stimulation.

The current model for the TCF regulatory switch is that ß-catenin promotes a dramatic change in transcriptional co-regulators associated with TCFs. Transducin-like enhancer of Split (TLE) co-repressors such as Groucho in flies [24] and UNC-37 in nematodes [29] bind to TCFs in the absence of signaling and recruit histone deacetylases, promoting gene silencing [7], [14]. ß-catenin binding to TCFs displaces these TLE proteins, somehow antagonizes other co-repressors, and in turn recruits several co-activators [7], [14].

In addition to this classic switch, many Wnt dependent targets in *C. elegans* utilize an additional mechanism, often referred to as the “Wnt/ß-catenin asymmetry” (WßA) pathway. WβA signaling promotes nuclear influx of the ß-catenin homolog SYS-1, while also promoting nuclear efflux of POP-1 [30]–[32]. This efflux of POP-1 requires the transforming-growth-factor- ß-activated kinase MOM-4, the Nemo-like kinase LIT-1 and the ß-catenin homolog WRM-1 [33]–[35]. WRM-1 acts with LIT-1 to phosphorylate POP-1, causing its nuclear export [36], which shifts the balance from POP-1 mediated repression to activation [30]–[32].

All TCFs contain a HMG domain, which recognizes a 9 bp consensus of SCTTTGATS (S = G/C) with high affinity [11], [37], [38]. In addition, most invertebrate TCFs and E-tail isoforms of vertebrate TCF1 and TCF4 contain a second DNA binding domain just downstream of the HMG domain called the C-clamp [11], [39]. This domain enables TCFs to recognize a second DNA motif, termed the Helper site, which is essential for the Wnt responsiveness of WREs in *Drosophila* and mammalian cell culture [39]–[41]. Functional Helper sites are often found near (<10 bp) functional HMG sites [39]–[41]. This supports a model where C-clamp containing TCFs recognize WREs through HMG domain-HMG site and C-clamp-Helper site interactions [9], [11].

In this study, we extended our analysis of Helper sites to three WREs directly regulated by the WßA pathway, from the *ceh-22*, *psa-3* and *end-1* loci. We found that all three had Helper site motifs near the functional HMG sites, and these Helper sites were crucial for expression of WRE reporters in transgenic worms. The presence of Helper sites dramatically increased binding of POP-1 to HMG sites *in vitro*. In some cases, multiple Helper sites functioned with a single HMG site to enhance POP-1 binding and mediate expression *in vivo*. An *in silico* search of the *C. elegans* genome for similar Helper sites-HMG site clusters uncovered a new WRE upstream of the *K08D12.3* gene, which is expressed in head muscles and the gut. In addition, a synthetic reporter containing concatamerized HMG and Helper sites displayed a novel POP-1 dependent pattern in the int9 cells of the larval intestine, leading us to discover that *pop-1* regulates the *C. elegans* defecation cycle. As previously reported [16], mutation of a single HMG site in the *end-1* WRE reporter results in derepression in MS blastomere, but this was not observed upon mutation of two nearby Helper sites, although these motifs were required for expression of the reporter in the E blastomere. This differential requirement of Helper sites in the transcriptional switch was also observed in a fly WRE. Consistent with the *cis*-regulatory mutagenesis data, we also found that the C-clamp is not required for TCF-mediated repression in the developing fly wing, but is essential for activation. These data support a model where basal repression of WREs occurs through HMG-HMG site interactions, whereas conversion of TCF to a transcriptional activator requires HMG-HMG site and C-clamp-Helper site recognition of DNA. Our data indicate that the TCF binding site is not passive during the transcriptional switch, but rather plays a more active role than previously suspected.

## Results

### Helper Sites Are Essential for POP-1 Regulation of the *ceh-22b* and *psa-3* WREs

**Figure 1:**
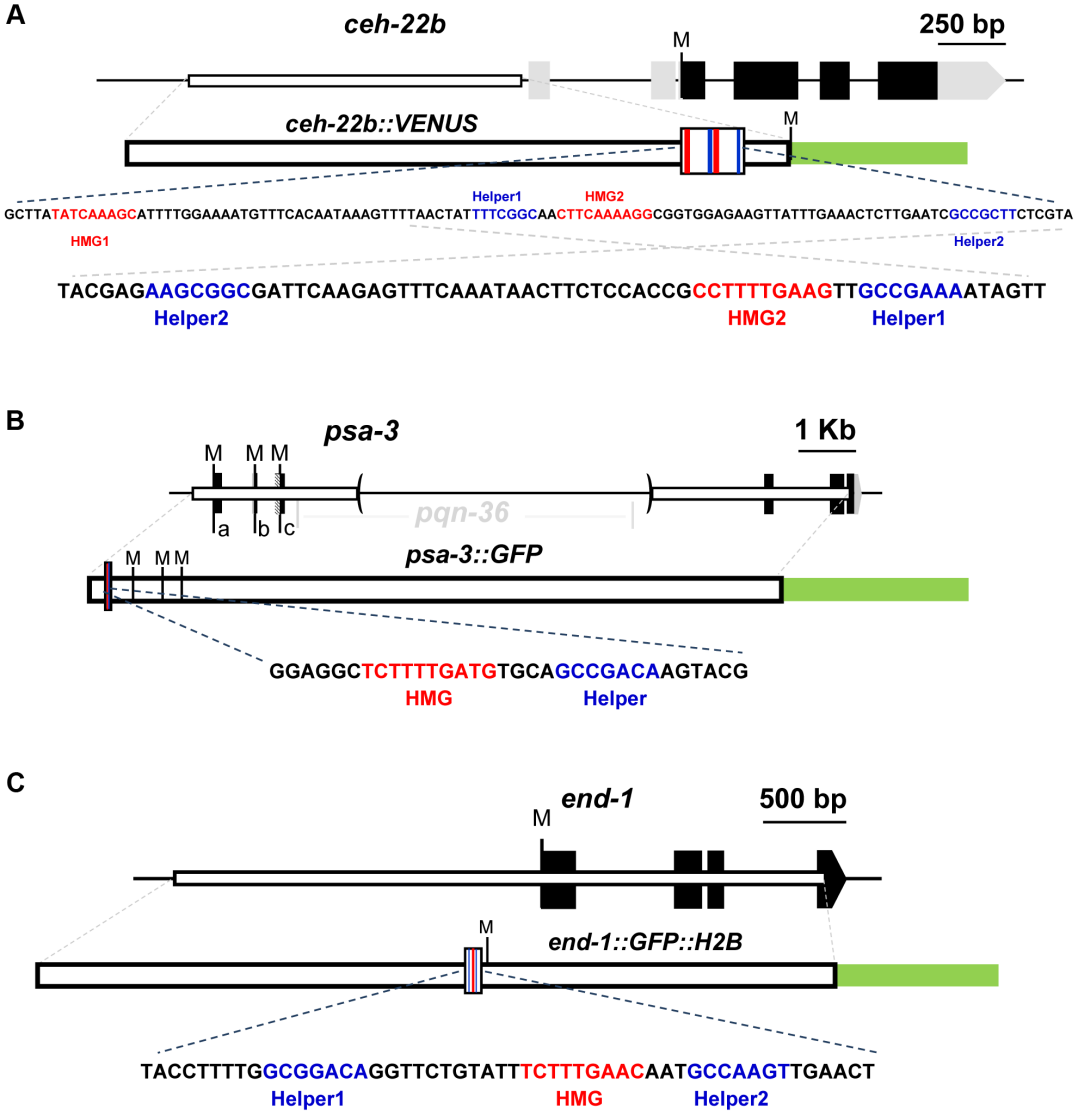
Schematics of the *ceh-22*, *psa-3* and *end-1* loci. For each locus, black boxes represent exons and gray boxes untranslated regions (UTRs). Start codons representing the Translation Start Site (TlSS) for each isoform are marked by ‘M’. White boxes represent the genomic region used to construct the WRE reporters and the green box the GFP variant used. The larger white boxes in the WRE reporter show the location of the HMG (red lines) and Helper sites (blue lines). Below each schematic are the genomic sequences highlighting the putative Helper sites (blue) and functional HMG sites (red) that were targeted for mutagenesis. (A) For the *ceh-22* gene (Gene ID: 179485), a transcriptional fusion of the *ceh-22b* isoform called *ceh-22b::VENUS* [42] was used for reporter analysis (nucleotides −1853 to −633 with the first nucleotide of the *ceh-22b* TlSS representing +1). (B) For *psa-3* (Gene ID: 181631), a translational fusion (*psa-3::GFP*) including promoter sequences (starting at -382) and the first exons of the a, b & c isoforms was used, where the *pqn-36* gene, located in the third intron was deleted, as indicated by the parentheses [43]. (C) For *end-1* (Gene ID: 179893), a translational fusion containing ∼2.2 kb of promoter sequence, known as *end-1::GFP::H2B* was used [16].

Like TCF/Pan and vertebrate TCF1E and TCF4E isoforms, POP-1 contains a C-clamp downstream of its HMG domain [9], [11]. Given the importance of Helper sites in fly and mammalian WREs that are regulated by C-clamp containing TCFs [39]–[41], we wanted to test the possibility that similar DNA motifs were present in *C. elegans* WREs. Therefore, we examined three targets that have previously been shown to be directly regulated by the WßA pathway, *ceh-22* [42], *psa-3* [43] and *end-1* [16] (Figure 1).

*ceh-22* encodes a homeodomain TF that is required for specification of the distal tip cells (DTCs) [42]. The DTCs play an essential role in gonadal arm elongation during development and in maintenance of the gonadal stem cell niche during adulthood [42], [44]. The *ceh-22* locus produces three isoforms, but the *ceh-22b/c* isoforms (termed *ceh-22b*) (Figure 1A) are sufficient to rescue gonadal defects in *ceh-22* mutants [42]. In hermaphrodites, a ∼1.2 kb transcriptional fusion upstream of the *ceh-22b* isoform (*ceh-22b::VENUS*) is expressed in the somatic gonadal precursors (SGPs) and their descendants Z1.a and Z4.p, the distal daughters of which become the DTCs [42]. Maintenance of this expression is dependent on *sys-1* and *pop-1*, and two HMG sites in the *ceh-22b::VENUS* reporter [42]. An examination of the sequences surrounding these functional HMG sites (termed HMG1 and HMG2) revealed the presence of two motifs (Helper1 and Helper2) similar to the Helper sites found in fly and mammalian systems (Figure 1A).

**Figure 2:**
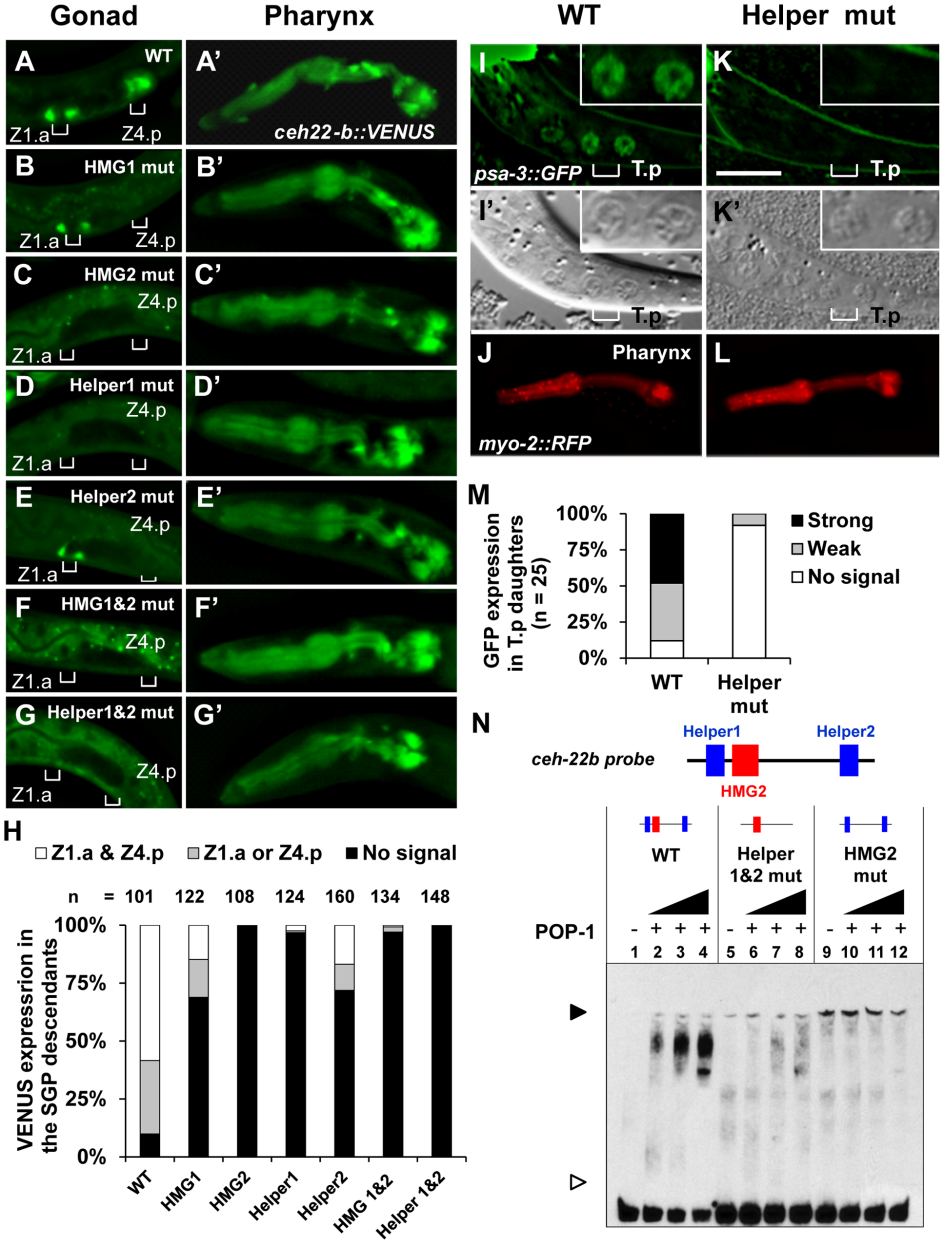
Helper sites are required for regulation of *ceh-22b* and *psa-3* WRE reporters. Deconvolved images of fixed L1 larvae showing the expression of stably integrated *ceh-22b::VENUS* post-division of distal SGP daughters Z1.a and Z4.p (A–G) and expression in the pharynx (A′–G′). VENUS expression in (A) Wildtype (WT), (B) HMG1 mutant, (C) HMG2 mutant, (D) Helper1 mutant, (E) Helper2 mutant, (F) HMG 1 & HMG2 mutant and (G) Helper1& Helper2 mutant were analyzed. Positions of the Z1.a & Z4.p daughter cells are indicated with brackets. (H) Semi-quantification of expression patterns of the different transgenic reporters, grouped three ways (expression in at least one daughter of both Z1.a & Z4.p cells; expression in at least one daughter in either Z1.a or Z4.p cells; no expression). Indicated individuals from three independent lines were examined for each construct except for the HMG1 & HMG2 double mutant construct where two lines were examined. Deconvolved (I & K) and Nomarski images (I′ & K′) of live L1 larvae with (I) Wildtype (WT) and (K) Helper mutant *psa-3::GFP* reporter. GFP expression was scored in the T cell granddaughters T.pa and T.pp which have the granular nuclear morphology of a neuroblast (I′ and K′, inset) [107]. *myo-2::RFP* was used as a coinjection marker. WT (J) and Helper mutant (L) animals with a similar RFP expression level in the pharynx were analyzed. (M) Semi-quantification of expression patterns in the T.p daughters of L1 larvae extrachromosomally expressing *psa-3::GFP* WT or Helper mutant reporter. (N) DNA binding of recombinant POP-1 to a 69 bp *ceh-22b* WRE probe (see Figure 1A for complete sequence) containing the HMG2 site and both Helper sequences was determined with EMSA. Wildtype probe (WT) shows a POP-1 dependent shift (lanes 1–4), while mutation of both Helper sites (lanes 5–8) or the HMG2 site (lanes 9–12) results in almost no detectable binding. The black arrowhead represents the DNA-protein complex and the white arrowhead represents unbound probe. The data shown are representative of more than three separate binding assays.

Mutagenesis of the HMG and Helper sites in the *ceh-22b::VENUS* reporter revealed that they all contribute to expression in SGP descendants. Transgenic worms expressing stably integrated versions of the wild type (WT) or mutant *ceh-22b::VENUS* reporters were generated (see Table S1 for details of the altered sequences). In addition to the SGP descendants during specific larval stages, *ceh-22b::VENUS* is also expressed in the pharynx (Figure 2A′) and this latter pattern is not dependent on *sys-1* and *pop-1* [42] . Therefore, we used pharyngeal expression as an internal control for transgene copy number, selecting lines with similar expression (Figures 2A′–2G′) to test the functionality of HMG and Helper sites. The WT reporter recapitulated the previously reported pattern of this WRE [42] in the Z1.a and Z4.p descendants in the late L1 hermaphrodites and subsequent stages (Figure 2A; data not shown). Mutation of HMG2 or Helper1 abolished the gonadal expression of this reporter (Figure 2C, 2D, 2H). Mutation of HMG1 or Helper2 resulted in a slightly less severe reduction (Figure 2H) with some animals expressing VENUS in the Z1.a or Z4.p daughters (Figure 2B, 2E). Simultaneous mutation of the two HMG or both Helper sites abolished gonadal expression (Figure 2F, 2G, 2H). These results indicated that Helper sites are required for activation of the *ceh-22b::VENUS* reporter in the Z1.a and Z4.p daughters.

To extend our analysis of Helper site function to another WßA pathway target, the *cis-*regulatory region of the Meis-related factor *psa-3* was examined. In hermaphrodites, POP-1 regulates the expression of *psa-3* in the posterior T-cell descendants, which give rise to the phasmid socket cells [43]. A translational *psa-3* fusion (Figure 1B), which can rescue the *psa-3* mutant phenotype [43], was used as the starting point to examine the functionality of a Helper site located near a HMG site (Figure 1B). This HMG site, located upstream of the *psa-3* Translational Start Site (TlSS; Figure 1B) was previously shown to be essential for expression of the translational reporter in the posterior T cell granddaughters T.pa and T.pp during the mid-L1 stage [43](Figure 2I). Wild-type (WT) and Helper mutant reporters (see Table S1 for base pair substitutions) were co-injected with the *myo-2::RFP* reporter, the pharyngeal expression of which was used as an internal control in the transgenic lines that were established (Figure 2J, 2L). Mutation of the Helper site abolished detectable expression of the *psa-3::GFP* reporter in the posterior granddaughters T.pa and T.pp in majority of transgenic larvae examined (Figure 2K, 2M). These data demonstrate that a Helper site near the functional HMG site plays a crucial role in activation of the WßA pathway target *psa-3*.

In analogy with fly and mammalian WREs [39]–[41], the Helper sites in the *ceh-22b* and *psa-3* reporters may enhance WßA signaling by increasing POP-1 binding. To test this, we performed Electrophoretic Mobility Shift Assays (EMSAs) with recombinant POP-1 and DNA probes containing Helper 1, HMG2 and Helper 2 from the *ceh-22b* reporter (Figure 1A) and the functional HMG and Helper sites from *psa-3::GFP* (Figure 1B). Both probes showed robust binding when incubated with POP-1 (Figure 2N; Figure S1). Mutation of the HMG2 site or Helper 1 & 2 sites dramatically reduced POP-1 binding to the *ceh-22b* probe (Figure 2N). Competition assays with unlabeled oligonucleotides demonstrated that Helper 1 & 2 sites are both significant contributors to POP-1 binding (Figure S1A). Similarly, the HMG and Helper site were both required to compete with labeled *psa-3* probe binding to POP-1 (Figure S1B). These results are consistent with a model where both HMG and Helper sites are required for high affinity binding of POP-1 to the *ceh-22b* and *psa-3* WREs.

### *In Silico* Identification of a *cis*-regulatory Element Upstream of the *K08D12.3/ZNF9* Locus Containing a Functional HMG-Helper Site Cluster

We next wanted to test whether a computational search for HMG and Helper site clusters could identify new POP-1 targets in the *C. elegans* genome. We utilized the open source algorithm Target Explorer [45], which we have used previously to detect novel WREs in *Drosophila* [40]. A position weight matrix for each motif was created based on the sequence of the functional HMG or Helper sites from *ceh-22b*, *psa-3* and *end-1* reporters plus the optimal HMG and Helper sites used in the synthetic reporter described below (Table S2). Because many *cis*-regulatory elements are located just upstream of nematode genes [46]–[49], we restricted the search to 500 bp regions upstream of every annotated TlSS or Transcriptional Start Site (TSS) in the *C. elegans* genome (24841 in total). Based on the organization of functional Helper and HMG sites in *ceh-22b*, *psa-3* and *end-1* (Figure 1), we utilized a search criterion where a 50 bp stretch had to contain at least two Helper sites and one HMG site to score positive. This generated a list of 115 clusters. 19 of these had the HMG site between the two Helper sites as found in the *ceh-22b* and *end-1* WREs (list provided in Table S3). Of this subgroup, three regions were selected for EMSA analysis with POP-1, based on the presence of more than two Helpers sites within 50 bp of the HMG site (*K08D12.3*) or the quality of the HMG and Helper sites (*RO5G6.5* and *Y66d12A.9*).

**Figure 3:**
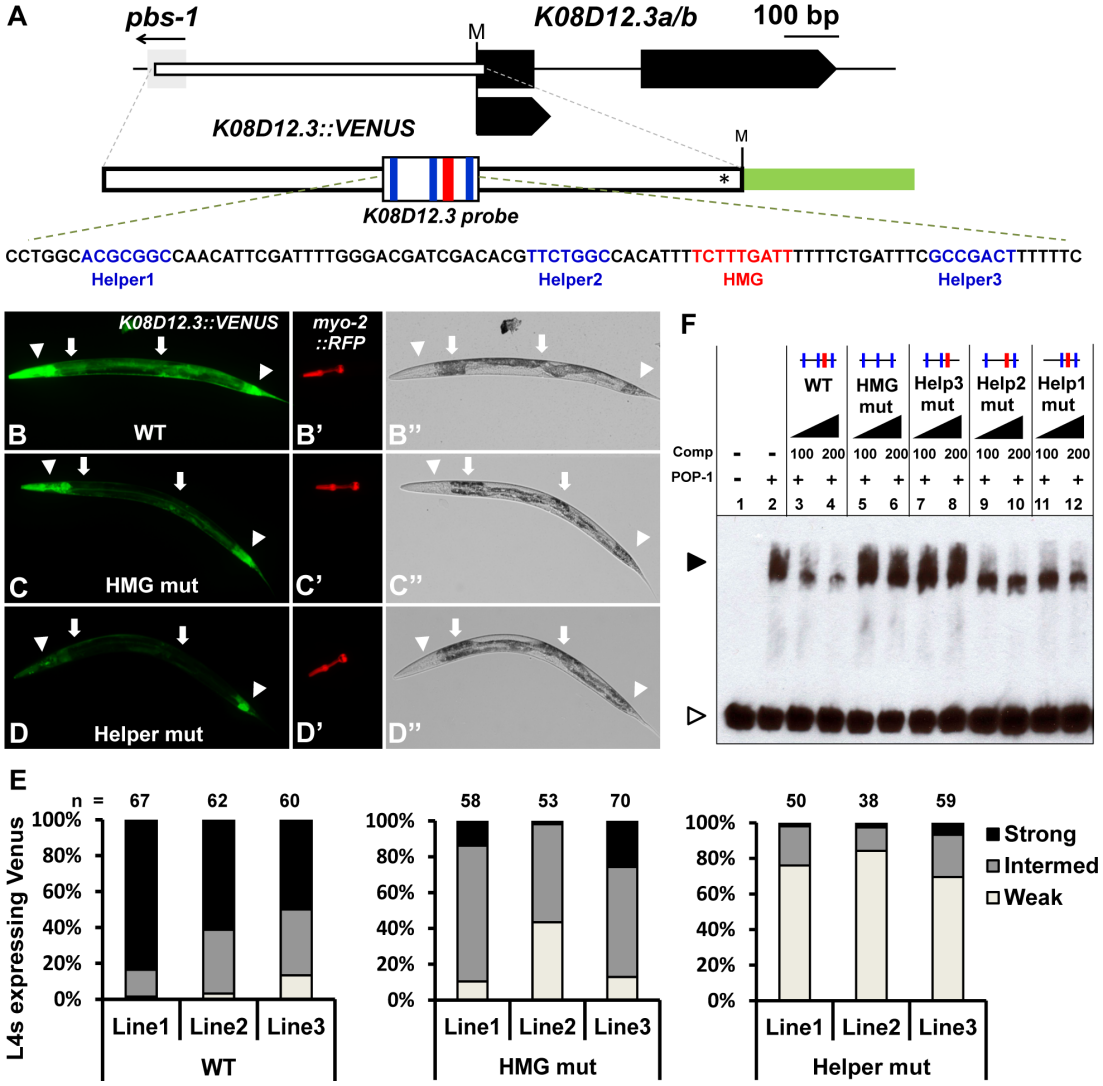
Identification of a new POP-1 target using a computational search for Helper site-HMG site clusters. (A) Schematic depicting the *K08D12.3* locus (Gene ID: 176979) with black boxes representing exons and the gray box the flanking gene *pbs-1*. The start codon is marked by ‘M’. The white box indicates the genomic region used to construct the GFP transcriptional reporter (nucleotides −579 to +14; first nucleotide of TlSS represents +1), with the asterisk indicating where the *K08D12.3* start codon was mutated to allow GFP to be read in the correct frame. The location of the HMG and Helper sites are indicated in red and blue respectively. Fluorescence (B–D; B′–D′) and Brightfield (B″–D″) images of live late L4 larvae extrachromosomally expressing the *K08D12.3::VENUS* reporter. Strong expression was seen in the head muscles, pharyngeal muscles, posterior intestine and hindgut (arrowheads) and moderate expression in the midgut (arrows). (B) Wildtype, (C) HMG mutant and (D) Helper mutant worms were scored based on the VENUS expression in the head muscles, pharyngeal muscles and intestine. (E) Histogram showing the expression analysis of late L4 larvae from three independent lines carrying either the WT, HMG mutant or Helper mutant *K08D12.3::VENUS* reporters, grouped into strong, intermediate or weak expressers, represented by the images in panels B, C & D, respectively. (F) Competition analysis using EMSA with POP-1 protein with a 90 bp probe (sequence shown in panel A) containing the three functional Helper sites and the functional HMG site from the *K08D12.3* WRE. The POP-1 dependent shift (lane 2) is competed by an excess of unlabeled WT probe (lanes 3, 4), while unlabeled HMG mutant probe (lanes 5, 6) or the Helper3 mutant probe (lanes 7, 8) does not compete even at 200 fold excess competitor levels. Unlabeled Helper1 mutant (lanes 9, 10) and Helper2 mutant (lanes 11, 12) probes displayed a moderate level of competition. The black arrowhead represents the DNA-protein complex and the white arrowhead represents unbound probe. The data is representative of three independent experiments.

Of the three Helper-HMG clusters tested, only the region upstream of the annotated *K08D12.3/ZNF9* gene had significant binding to POP-1 (Figure S2). The original *K08D12.3* probe contained three Helper sites and one HMG site. A shorter probe missing one of the Helper sites was still bound by POP-1, but to a lesser degree (Figure S2), suggesting that Helper 1 was required for maximal POP-1 binding. Indeed, competition experiments indicated that the HMG site and all three Helper sites were required for POP-1 binding, with the HMG and Helper3 sites having the greatest contribution (Figure 3F). Therefore, all four sites were considered in our *in vivo* analysis.

To test whether the region upstream of *K08D12.3/ZNF9* could drive expression of a reporter gene, a 585 bp stretch upstream of the TlSS was cloned behind a VENUS reporter (Figure 3A), and tested for expression *in vivo*. Strong reporter expression was observed in the foregut, anterior body wall musculature (dorsal and ventral head muscles), posterior intestine and hindgut during larval stages till adulthood (Figure 3B, 3B″; data not shown). Weaker expression was also seen in the midgut, mid-body wall musculature and the posterior body wall muscles during these stages (Figure 3B, 3B″; data not shown). This expression pattern was similar (but less intense) than a previously reported pattern of a ∼2.3 kb *K08D12.3::GFP* transcriptional fusion [47], [48]. No decrease in the *K08D12.3* reporter was observed in *pop-1 (q645)* mutants or animals depleted of POP-1 via RNAi feeding (data not shown). However, these conditions only partially reduce *pop-1* gene activity [50], [51] and are therefore inconclusive.

To provide a more definitive test of POP-1 regulation of the *K08D12.3* reporter, late L4 hermaphrodites containing a wild-type, a HMG mutant or a triple Helper mutant reporter were scored based on VENUS expression in the hindgut and midgut, as well as the head and pharyngeal muscles, and characterized as having strong, intermediate or weak expression (Figure 3B–E). As with the *psa-3* reporter, *myo-2::RFP* was used as an internal control (Figure 3B′–3D′). Mutation of the HMG site and Helper sites significantly affected VENUS expression in the head muscles, midgut, and foregut with less reduction in hindgut expression (Figure 3B–E). The Helper site mutant lines had less expression than the HMG site mutants, most notably in the pharyngeal muscles (Figure 3C–E). These data suggest that the *K08D12.3* reporter is a direct target of POP-1.

### The C-Clamp of POP-1 Is Required for Enhanced Binding to HMG-Helper Site Clusters

**Figure 4:**
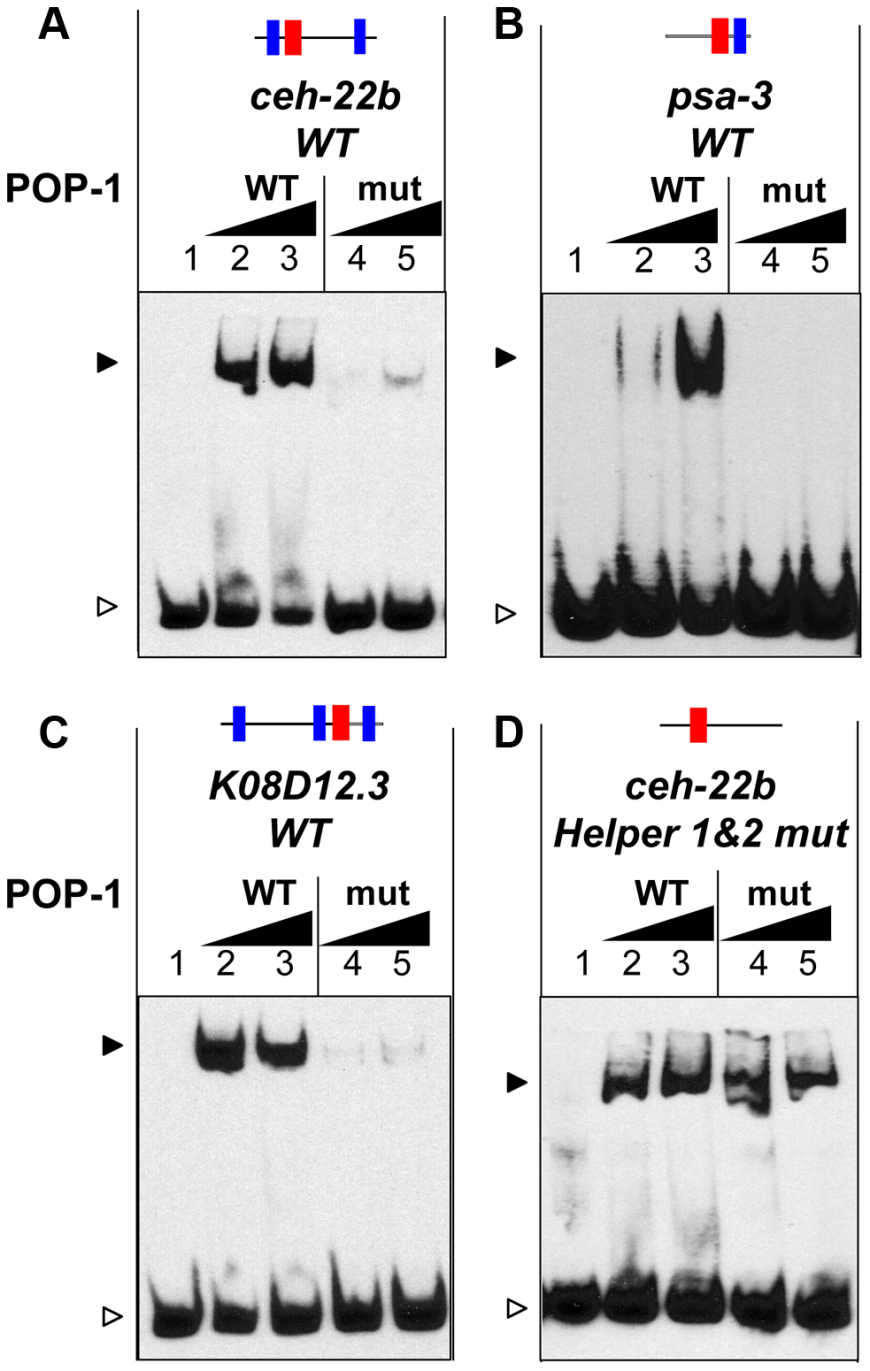
The C-clamp of POP-1 facilitates binding to DNA containing Helper sites. (A–D) EMSAs showing binding of wild-type recombinant POP-1 and a POP-1 C-clamp mutant to the *ceh-22b* WRE probe (1.5 femtomoles/reaction) described in Figure 1A and 2N (A), the *psa-*3 probe (3 femtomoles/reaction) described in Figure 1B and S1B (B), the *K08D12.3* probe (4 femtomoles/reaction) described in Figure 3A and 3F (C) and the *ceh-22b* probe (3 femtomoles/reaction) with both Helper sites mutated (D). The *ceh-22b*, *psa-3* and *K08D12.3* WT probes show strong binding with increasing amounts (0.4 and 0.8 µg/reaction) of POP-1 WT protein (lanes 2 and lane 3 respectively) but not with the POP-1 C-clamp mutant (lane 4 and lane 5 respectively). Under conditions designed to detect lower affinity binding (0.75 and 1.5 µg of POP-1; 3 femtomoles of probe and longer exposure times), binding to the *ceh-22b* probe lacking Helper sites (containing only the HMG2 site) was similar with WT and mutant POP-1. The data are representative of three independent experiments.

The finding that Helper sites are important for high affinity POP-1 binding (Figure 2N) suggests that the C-clamp of POP-1 is required for this binding. To test this, recombinant POP-1 containing two substitutions in the C-clamp (K365A & R367E) was expressed and purified. The corresponding mutations in TCF/Pan abolish DNA binding to Helper site DNA (A. Ravindranath and K. M. Cadigan, unpublished). This C-clamp mutant displayed a dramatic reduction in binding to a *ceh-22b* HMG-Helper site probe (Figure 4A). Mutation of the C-clamp also dramatically reduced affinity of POP-1 for HMG-Helper site containing probes from the *psa-3* and *K08D12.3* loci (Figure 4B, 4C). These data demonstrate that high affinity binding of POP-1 to functionally important WRE DNA requires the C-clamp domain.

As described above, Helper sites are required for high affinity binding to DNA by POP-1 (Figure 2N, Figure 3F, Figure S1). This requirement is not altered by mutation of the C-clamp (Figure S3). However, POP-1 can still recognize HMG site DNA in the absence of Helper motifs or a functional C-clamp, albeit at lower affinity. Under conditions allowing the detection of weaker binding, i.e., increased probe concentration and longer exposure times, wild-type and C-clamp mutant POP-1 showed comparable binding to a *ceh-22b* probe where the Helper sites are mutated (Figure 4D; see also Figure S3, which uses conditions that only detect higher affinity binding). These data demonstrate that the HMG domain of POP-1 can recognize HMG site DNA, but higher affinity binding required a bipartite mechanism similar to what we previously reported for TCF/Pan [40].

### A Synthetic Reporter Containing HMG-Helper Sequences Reveals a POP-1 Dependent Pattern in int9 Intestinal Cells

Synthetic reporters containing concatemerized high-affinity HMG sites have been used as Wnt/ß-catenin signaling readouts in several systems [52], e.g, TOPFLASH in mammalian cell culture [53] and TOPGAL in transgenic mice [54]. Similar HMG site reporters do not work well in *Drosophila* [40], but multiple HMG-Helper site pairs provide a much more sensitive indicator of Wnt/ß-catenin signaling [40]. In *C. elegans*, a reporter known as POPTOP (POP-1and TCF Optimal Promoter) contains seven copies of a high affinity HMG site, which displays a wide range of expression including several cells where Wnt/POP-1 signaling is known to occur [55]. To test whether Helper sites can improve the sensitivity or selectivity of HMG sites in this synthetic context, we constructed a reporter containing six HMG-Helper site pairs (see Materials and Methods for sequence) called POPHHOP (POP-1 and HMG-Helper Optimal Promoter) and tested it for expression in *C. elegans*.

**Figure 5:**
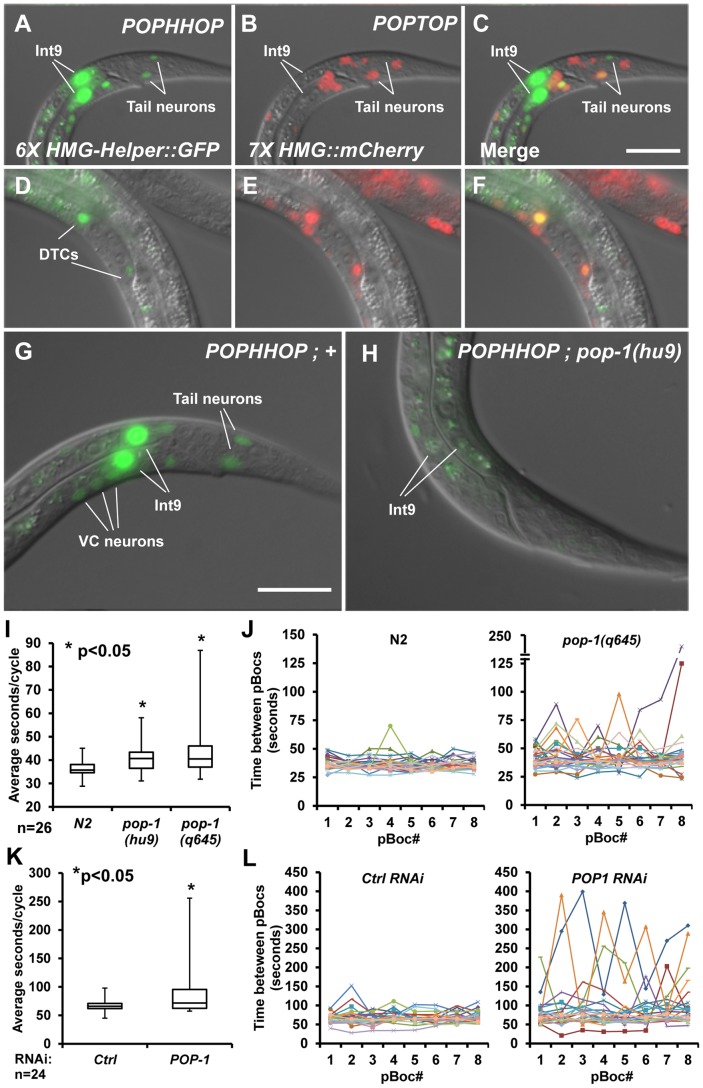
A synthetic HMG-Helper site reporter reveals a novel POP-1 function in rhythmic defecation behaviour. (A–F) Nomarski images of animals with stably integrated *POPHHOP* (*6× HMG-Helper::GFP*) and *POPTOP* (*7× HMG::mCherry*) reporters showing GFP (A, D) and mCherry (B, E) fluorescence. Live L1 larvae have overlapping expression of GFP and mCherry in some tail neurons (A–C) and live L3 larvae display overlapping DTC expression (D–F). In addition, POPHHOP displayed strong expression in the int9 intestinal cells of early L1 Larvae (A) onward through adulthood (not shown). (G–H) Stably integrated *POPHHOP* animals in a wild-type (G) or *pop-1(hu9)* background (H). The reporter expression seen in the int9 cells, tail neurons, and occasionally in the VC neurons is low or undetectable in the *pop-1* mutants. Scale bars = 10 µm. (I) Box-whisker plot showing the median (line inside the box), third quartile (upper box), first quartile (lower box), longest pBoc cycle time (upper whisker limit) and shortest pBoc cycle time (lower whisker limit) for N2 controls and two *pop-1* alleles at the L2 stage. A statistically significant increase was seen in the pBoc cycle time based on a Student's two-tailed *t* test (see Table 1). (J) 8 individual pBocs (X-axis) were monitored (n = 26, each color representing one larva) for each genotype and plotted against time between each pBoc (y-axis). *pop-1(q645)* mutants have greater variability between pBocs than the wild-type N2 control. (K) Box-whisker plot showing the pBoc period of *pop-1* depleted worms compared to ctrl RNAi worms using the OLB11 strain, which allows intestine-specific RNAi [65], [66]. Animals were assayed at the young adult stage. A statistically significant increase was seen in the pBoc cycle time based on a Student's two-tailed *t* test (see Table 1). (L) 8 individual pBocs (X-axis) were monitored in young adults (n = 24, each color representing one adult) for each genotype and plotted against time between each pBoc (y-axis). *pop-1 RNAi* leads to a high variability in the cycle time in *pop-1* depleted adults compared to controls.

Similar to POPTOP, stably integrated POPHHOP was expressed in several cells where Wnt/ß-catenin signaling is known to be active (Figure 5D; Figure S4). The POPHHOP reporter was active in cells not previously known to receive Wnt signals, e.g., unidentified tail neurons and posterior cells in the ventral nerve cord during the early L1 stage (Figure 5A; Figure S4A). Expression was also seen in seam cell nuclei, muscle nuclei along the anterior/posterior axis and the QL.d nuclei (Figure S4C–G). The most prominent novel pattern observed with POPHHOP was in the posterior most intestinal cells known as ‘int9 cells’, during the early L1 stage (Figure 5A, 5C, 5G; Figure S4E, S4F) onward to adults (data not shown). This pattern was lost in a genetic background homozygous for the hypomorphic allele *pop-1(hu-9)* (Figure 5H). In sum, the inclusion of Helper sites in a HMG site synthetic reporter altered the specificity of reporter expression, and should be a useful tool to study POP-1 readouts in *C. elegans*.

### The Role of POP-1 in the *C. elegans* Defecation Cycle

The expression pattern of POPHHOP in int9 cells was intriguing, given the central role that these cells are known to play in regulating the defecation cycle in *C. elegans* [56]. The cycle starts with contractions in the posterior body wall muscles (pBoc) at regular (45–50 sec) second intervals in adult worms [57]. Each pBoc is followed by anterior body wall contractions (aBoc) and expulsion (Exp) of the fecal content completing a defecation cycle [57]. The intestine acts as a pacemaker where calcium oscillations set the period of each cycle [56], [58]–[60]. Calcium spikes originate in the int9 cells, which initiate the pBoc step [56]. POP-1 is expressed in these cells [61] and is required for POPHHOP expression in int9 cells (Figure 5H). This suggested that POP-1 could play a role in regulating rhythmic defecation in *C. elegans*.

Strong alleles of *pop-1* are early larval or embryonic lethal [19], [50], [62]. Therefore, two hypomorphic alleles of *pop-1*, both containing single amino acid substitutions in the ß-catenin binding domain, were used to examine the role of POP-1 in the defecation cycle. *pop-1(hu9)* homozygotes survive to adulthood [63], [64], while a fraction of hermaphrodites homozygous for the stronger allele *pop-1(q645)* make it to adulthood [50]. Since POPHHOP was regulated by POP-1 at the early L1 stage and onward (Figure 5H; data not shown), we examined pBoc cycle lengths and expulsion events in control N2 animals and the hypomorphic *pop-1* mutants in early L2 larvae, well before any lethality was apparent in the *pop-1(q645)* animals.

We found that control N2 animals have a highly regular defecation cycle at the L2 stage, displaying a mean periodicity of 36.4 seconds between pBocs with a standard deviation of 3.5 seconds. L2 larvae homozygous for the *pop-1(hu9)* and *pop-1(q645)* alleles had a 11–18% increase in mean cycle time and greater arrhythmia than N2 controls (Table 1). The increased arrhythmia can be observed when individual cycle times are plotted for each background (Figure 5J) and in a box-whisker plot (Figure 5I). In addition, the expulsion of fecal content, which is tightly coupled to the pBoc in each cycle, failed to occur 6.7% of the time in the *pop-1(q645)* mutants, while such failures were never seen in control or *pop-1(hu9)* animals (Table 1). Taken together, our analysis of the expression pattern of the POPHHOP reporter led to the discovery of a previously unsuspected requirement for POP-1 in regulating the cycle time and rhythmicity of *C. elegans* defecation.

**Table 1:** Reduction of *pop-1* gene activity results in a prolonged defecation cycle.

Strain	n	pBOC periodicity mean ± SD	Total # pBocs observed	% missed expulsions
N2	26	36.4±3.5	208	0.0
*pop-1(hu9)*	26	40.6±6.1**	208	0.0
*pop-1(q645)*	26	42.9±10.8**	208	6.7
N2 (ctrl RNAi)	12	49.8±3.4	96	0.0
N2 (*pop-1* RNAi)	12	54.2±5.1*	96	0.0
OLB11 (ctrl RNAi)	24	67.5±10.8	192	1.0
OLB11 (*pop-1* RNAi)	24	92.4±49.5*	192	13.0

Eight pBocs and expulsions were observed for each individual, with the N2 and pop-1 mutants assayed at the L2 larval stage, and the RNAi fed individuals assayed as young adults.

* P<0.05;

** P<0.01.

Although the *pop-1* alleles used above have no obvious anatomical defects at the L2 stage and survive to adulthood, the possibility remains that the effect on defecation in these mutants was indirectly due to abnormal embryonic development. To address this, RNAi feeding was performed with L1 larvae, with defecation measured in young adults (0–12 hours). RNAi depletion of *pop-1* resulted in a significant increase (8.8% in the mean period) in the defecation cycle and greater variability compared with control fed animals (Table 1 and data not shown). When *pop-1* depletion was limited to intestinal cells, using strain OLB11, which is mutant for the Argonaute gene *rde-1* and contains a transgene expressing *rde-1* under the control of the *elt-2* intestinal promoter [65], [66], a more dramatic effect on defecation was observed (36.9% increase in the mean period) with a concomitant increase in variation in cycle length (Figure 5K, 5L). The frequency of missed expulsions following pBoc was 13 times higher than controls (Table 1). These results indicate that the requirement for POP-1 in regulating the defecation cycle resides in intestinal cells.

### Helper Sites Are Required for Activation of the *end-1* WRE, but Not Basal Repression by POP-1

Although the TCF transcriptional switch is the prevailing model for Wnt/β-catenin gene regulation, for many WREs, there is little evidence for repression in the absence of signaling [9]. For example, there is a dramatic loss of activation when HMG sites are mutated in the *ceh-22b*, *psa-3* and *K08D12.3* reporters discussed thus far, but no detectable derepression. This is likely due to a lack of local activators in these elements that could drive expression in the absence of POP-1 mediated repression [1], [9]. To address a possible role for Helper sites in POP-1 basal repression, we examined the WRE regulating the GATA factor *end-1* (Figure 1C). In early embryogenesis, the WßA pathway is required for maximal expression of *end-1* in the endodermal descendant of EMS, known as E cells [15], [16], [18]. In the other EMS derived daughter cell, the mesodermal MS, POP-1 represses *end-1* expression [15]–[20]. Consistent with this, mutation of a single HMG site in an *end-1* translational fusion (*end-1::GFP::H2B*) causes a reduction in expression in E cells, accompanied by a dramatic elevation of expression in MS cells [16]. Two putative Helper sites are located near this functional HMG site (Figure 1C). Thus, the bimodal regulation of *end-1* by POP-1 provides an ideal system in which to test the role of Helper sites in a target where both sides of the transcriptional switch are robustly apparent.

**Figure 6:**
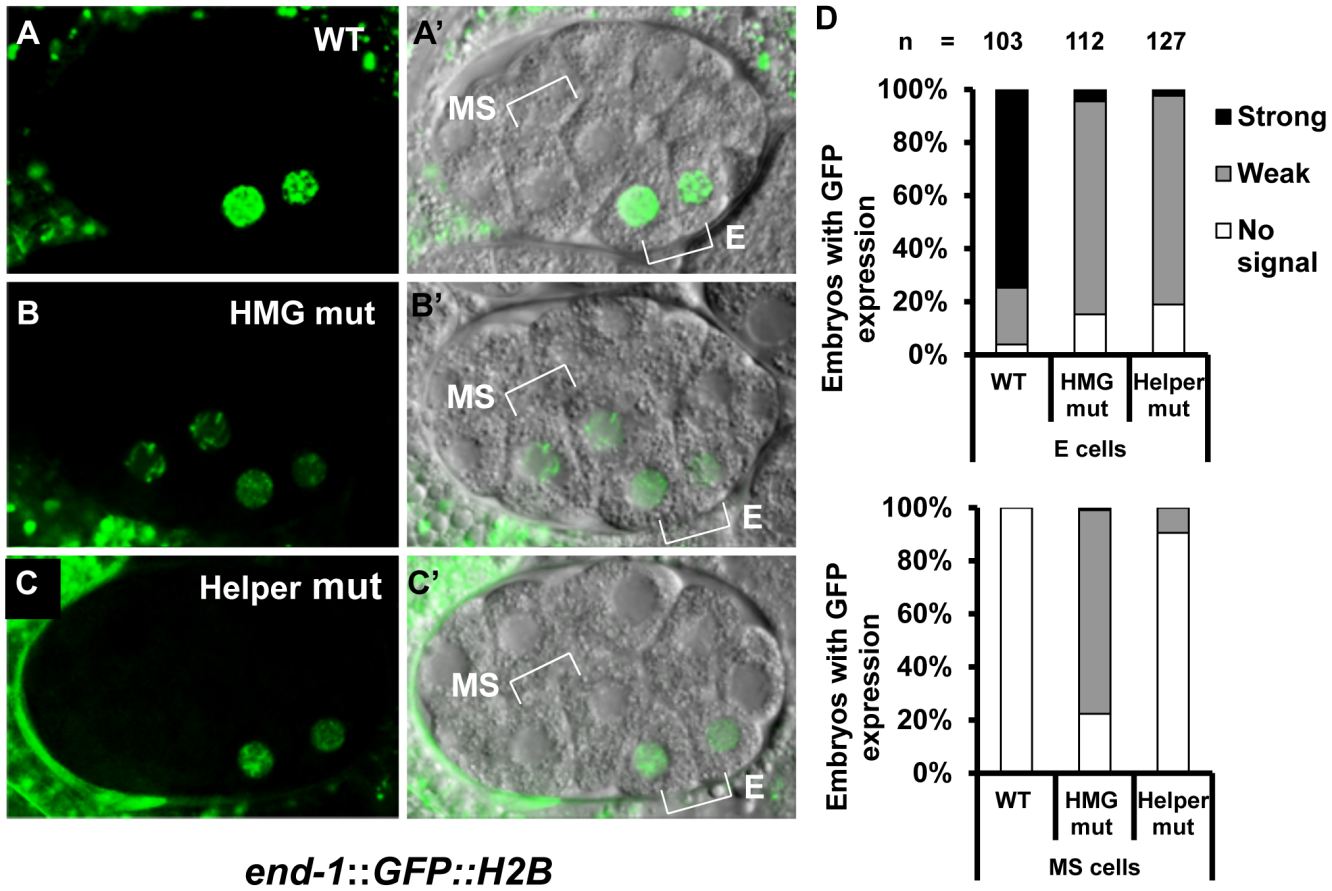
HMG and Helper sites contribute differentially to the regulation of *end-1* during early embryogenesis. Deconvolved (A–C) and Nomarski (A′–C′) images showing expression of a stably integrated *end-1::GFP::H2B* reporter in the endodermal (E) and/or mesodermal (MS) daughters of live embryos at the 2E stage. The wild type (WT) reporter shows strong GFP expression in the E cell daughters (A, A′). Mutation of the HMG site leads to a significant reduction of GFP expression in the E daughters and a significant derepression of *end-1::GFP::H2B* in the MS daughters (B, B′). Mutation of two Helper sites leads to a significant reduction of GFP in the E daughters (C, C′), but little or no depression in the MS daughters (C, C′). (D) Histograms summarizing the results from over 100 embryos from three independent lines for each construct, grouped by strong, weak or no expression in the E (upper graph) and MS (lower graph) cells.

Transgenic worms expressing stably integrated WT or mutant *end-1::GFP::H2B* reporter fusions were generated (Figure 1C; Table S1) and examined at the 2E stage. As previously reported [16], the WT reporter had a strong GFP expression in the two E cell daughters while mutation of the HMG site led to a strong decrease of expression in these cells, and ectopic expression in the MS descendants with high penetrance (Figure 6A, 6B, 6D). Mutation of the two putative Helper sites resulted in the same dramatic decrease in E cell expression as seen in the HMG site mutant (Figure 6C, 6D). However, mutation of the Helper sites resulted in almost no detectable derepression in the MS cells (Figure 6C, 6D). These data indicated that the Helper sites are largely dispensable for repression of *end-1* in the MS cells, while they are required for its maximal activation in the E cells.

### The Differential Requirement of HMG and Helper sites in the Transcriptional TCF Switch Is Conserved in Flies

**Figure 7:**
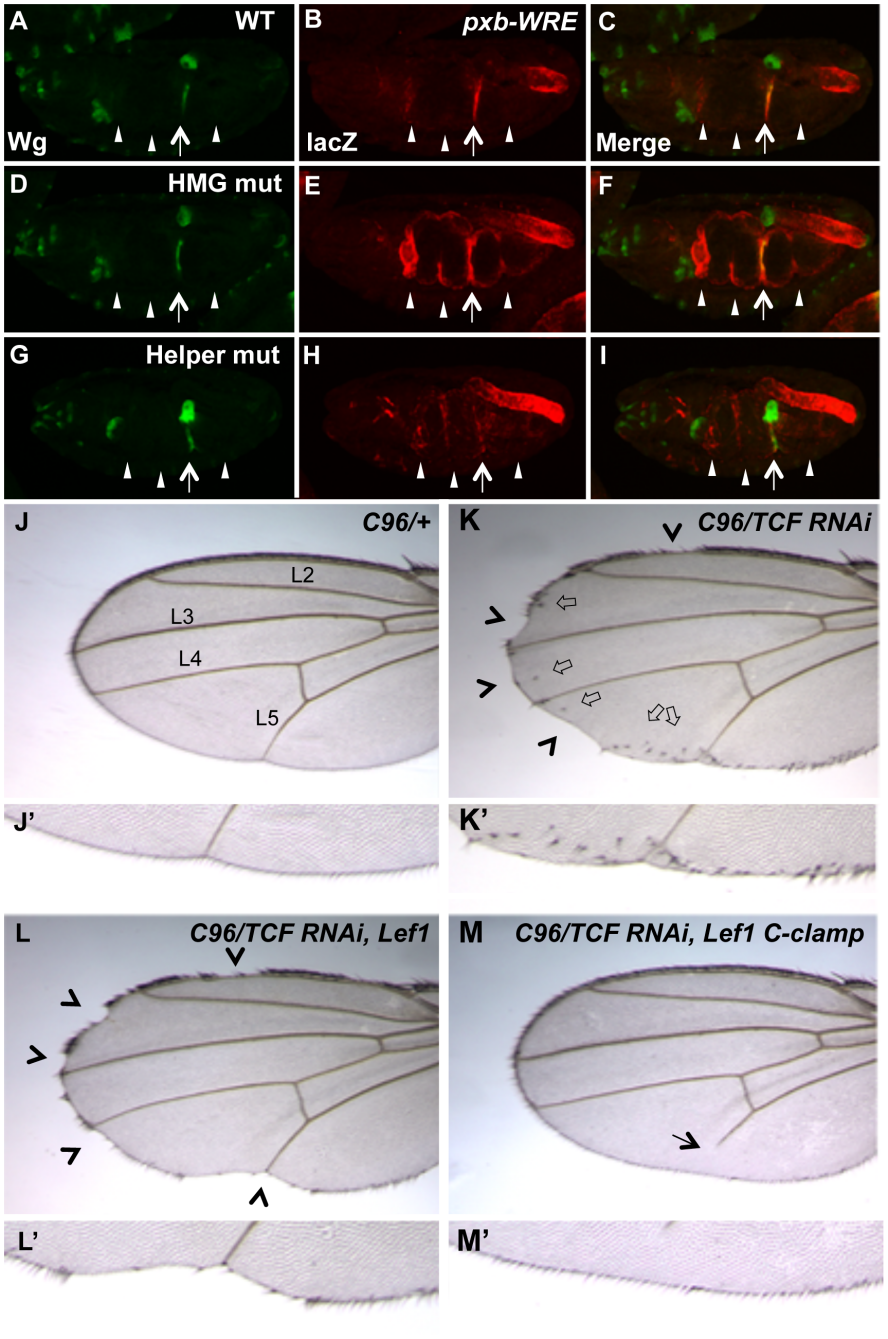
Helper sites and the C-clamp are not required for basal repression of Wg targets in *Drosophila*. (A–I) Confocal images of stage 16–17 embryos containing a *pxb::lacZ* WRE reporter immunostained for Wg (green) (A, D & G), lacZ (red) (B, E & H) or merged (C, F & I). The wild-type reporter shows a pattern overlapping with Wg in the second constriction of the midgut, and a non-overlapping pattern in the hindgut (A–C). Mutation of two HMG sites leads to a strong depression through the entire midgut (arrowheads), without affecting lacZ expression in the second constriction (arrow) (D–F). Mutation of two Helper sites leads to a significant decrease in the lacZ expression in the second constriction (arrow) with weak ectopic expression (arrowheads)(G–I). The hindgut expression did not vary in the different constructs and was used as an internal control. All images are representative of at least 20 embryos. (J–M) Images of adult wings containing the wing driver *C96-Gal4* crossed to wildtype (WT) (J, J′), UAS-TCF/Pan RNAi (K, K′) or UAS-TCF/Pan RNAi plus UAS-LEF1 (L, L′) or UAS-LEF1 plus the C-clamp of TCF/Pan (M, M′). Knockdown of TCF/Pan leads to notches (arrowheads) and ectopic wing margin bristles (block arrows) along the periphery of the wing (where *C96-Gal4* is active; K, K′). Expression of the human LEF1 transgene significantly rescues the ectopic bristle expression, but not the notches (L, L′). Expression of a LEF1-C-clamp chimera rescues the wing margin defects and prevents ectopic bristle formation, and causes a L5 vein defect (arrow). Details about the penetrance of these phenotypes are listed in Table 1.

In our previous report on the role of Helper sites in *Drosophila*, the four WREs tested in transgenic reporter assays were similar to the *ceh-22b* and *psa-3* reporters, i.e., they are strongly activated by Wnt/ß-catenin signaling but have little detectable derepression when their HMG sites are removed [40]. However, we have found a WRE upstream of the *pxb* gene (Gene ID: 41899), originally identified through a computational search for HMG-Helper site clusters in the fly genome [40], that has a significant basal repression component. When this WRE was placed upstream of a minimal promoter driving lacZ and inserted into the fly genome through P-element transgenesis, expression was observed in a pattern that overlapped with Wingless (Wg, a fly Wnt) in the second constriction of the embryonic midgut (Figure 7A–C; see arrow). The *pxb-*WRE reporter was also expressed at low levels in the anterior midgut (Figure 7B, first arrowhead) and in the hindgut where there is no detectable Wg expression (Figures 7A–C). Mutation of two HMG sites in the *pxb*-WRE resulted in a strong derepression throughout the midgut (Figure 7E, arrow and arrowheads) and no change in the hindgut expression (Figure 7E). These results indicate that in the embryonic midgut, there is a large degree of basal repression of the *pxb*-WRE by TCF/Pan.

In contrast to the HMG sites, mutation of two nearby Helper sites caused a moderate decrease of *pxb*-WRE expression in the second midgut constriction (Figure 7H, arrow), with only mild ectopic expression in the midgut (Figure 7H, arrowheads). This derepression was extremely weak compared to that seen in the HMG mutant embryos (Figure 7E, 7H). In both mutant constructs, expression in the hindgut was not significantly affected, serving as an internal control (Figure 7B, 7E, 7H). These data indicate that the Helper sites are required for activation of *pxb*-WRE by Wg signaling but are largely dispensable for repression of this reporter in cells with no detectable Wg expression.

If Helper sites are primarily required for TCF/ß-catenin activation of gene expression and not basal repression by TCF, is the C-clamp domain of TCFs only required for the activation side of the transcriptional switch? To test this idea, a TCF/Pan rescue assay was established in the developing fly wing. Wg signaling is required for specification of the wing margin and adjacent sensory bristles, with loss of signaling resulting in notches in the wing blade [67], [68] and ectopic signaling causing ectopic sensory bristles [69], [70]. When TCF/Pan was depleted in flies containing the wing margin specific Gal4 driver C96 [71] and a UAS-TCF/Pan RNAi construct [72], notches along most of the distal margin were observed with 100% penetrance (Figure 7K, 7K′; Table 2). In addition, a large number of ectopic wing margin bristles (∼22/wing) were seen (Figure 7K, 7K′; Table 2), likely due to derepression of the Wg targets specifying sensory bristles.

**Table 2:** The C-clamp is required for Wg activation but not basal repression in a TCF/Pan rescue assay.

*C96-Gal4* crossed to: (n)	Notches (%)			Ectopic bristles/wing (n)	L5 vein defect (%)*
	None	Small	Large		
+ (46)	100			0 (20)	0
Lef1 A (38)	100			0 (20)	5.2
Lef1 B (43)	95.3	4.7		0 (20)	4.7
Lef1-C-clamp A (38)	100			0 (20)	84.2
Lef-1-C-clamp B (39)	100			0 (20)	87.2
TCF/Pan-RNAi (46)		2.2	97.8	22.6 (20)	47.8
Lef1 A; TCF/Pan-RNAi (60)		8.3	91.7	3 (30)	3.3
Lef1 B; TCF/Pan-RNAi (52)		7.7	92.3	0.63 (30)	11.5
Lef1-C-clamp A; TCF/Pan-RNAi (46)	71.7	26.0	2.3	1.7 (20)	97.9
Lef-1-C-clamp B; TCF/Pan-RNAi (38)	100			0 (20)	55.3

Two independent lines of UAS-Lef1 and UAS-Lef1-C-clamp with similar expression levels (see Figure S5B) were assayed. Expression of either transgene with the *C96-Gal4* driver had little or no effect on wing development in an otherwise wild-type background. Percentages tabulated for the wing phenotypes seen upon knock down of TCF. Depletion of TCF/Pan with a UAS-driven RNAi hairpin causes mostly large notches, and leads to more than 20 ectopic bristles per wing and a high penetrance of L5 vein defects. Expression of human Lef1 (Lef1) significantly rescues the ectopic bristles, but has little effect on the size and frequency of the wing notches. In contrast, expression of Lef1 with the C-clamp of TCF/Pan (Lef1-C-clamp) rescues both ectopic bristles and the wing notch phenotype. (n) represents the number of wings examined for each genotype. Depletion of TCF/Pan and expression of Lef1 and Lef1-C-clamp also resulted in a disruption of the L5 vein (see Figure 7M and data not shown). Since this phenotype has not been linked to Wg signaling, it is not considered further in this report.

The TCF/Pan RNAi phenotypes were used to assay the ability of UAS transgenes expressing the human TCF family member LEF1 (which contains a HMG domain but no C-clamp), or LEF1 with the C-clamp of TCF/Pan (LEF1-C-clamp; see Figure S5A for description of LEF1 proteins), to rescue the derepression and loss of activation phenotypes in C96::TCF/Pan RNAi wings. A human TCF was used in this assay because it is insensitive to the UAS-TCF/Pan RNA hairpin, which targets the ORF of endogenous *TCF/Pan* mRNA.

Both the LEF1 and LEF1-C-clamp transgenes had biological activity in the fly wing, but with dramatically different specificities. LEF1 was unable to rescue the wing notch phenotype (i.e., activation) but strongly suppressed the formation of ectopic bristles (basal repression) (Figure 7L, 7L′; Table 2). In contrast, the LEF1-C-clamp chimera was able to rescue both the notch and bristle phenotypes (Figure 7M, 7M′; Table 2). More than a dozen independent UAS-LEF1 and UAS-LEF1-C-clamp lines were generated, and the ones at the lower end of the expression spectrum were used in this rescue experiment, because higher expression of either transgene caused wing notches in an otherwise wild-type background (data not shown). We suspect that too much of either LEF1 protein inhibits Wg signaling by titrating out fly ß-catenin in the nucleus, in analogy to high nuclear POP-1 levels in the WßA pathway [18], [73]. Western blot analysis revealed that the LEF1 and LEF1-C-clamp transgenes used for the rescue were expressed at similar levels (Figure S5B). These data fit with the *cis*-regulatory mutagenesis of the *end-1* and *pxb* WREs (Figure 6; Figure 7A–7I), supporting a model where basal repression by TCFs involves HMG domain-HMG site interactions, while ß-catenin dependent activation requires HMG domain-HMG site and C-clamp-Helper site binding.

## Discussion

### Helper Sites and POP-1 Recognition of *C. elegans* WREs

In this report, we demonstrate that Helper sites enhance POP-1's ability to bind to DNA with high affinity and are critical for the expression of four distinct *C. elegans* WREs expressed in a variety of tissues and developmental stages (Figure 2; Figure 3; Figure 6). In this respect, *C. elegans* is similar to *Drosophila*, where we have previously shown that Helper sites are just as important as HMG sites for WRE activity in vivo [40]. HMG and Helper sites are also equally important for activation of specific WREs in mammalian cell culture by TCF1 and TCF4 isoforms containing a C-clamp [41]. Since we have found functional Helper sites in every fly and worm WRE that we have rigorously characterized (a total of ten), it is tempting to suggest that many more WREs in these organisms utilize a similar HMG-Helper site mechanism. We also suggest that in other invertebrate phyla, such as porifera, cnidarians and echinoderms, which possess one *TCF* gene with a C-clamp [74]–[76], TCFs likely recognize WREs through a similar bipartite mechanism to perform many of their essential functions.

While Helper sites are clearly important for several fly, nematode and mammalian WREs [40], [41; this report], it is also true that multimerized HMG sites are sufficient for Wnt responsiveness in reporters such as TOPFLASH and POPTOP [52]–[54]. However, such high density clusters (typically 4–7 are used) of perfect consensus HMG sites are not found in endogenous WREs [9], [11], [52]. While these synthetic reporters are useful tools for studying Wnt signaling in many systems, they do not reflect the situation in naturally occurring WREs, where the lower density and degeneracy of HMG sites requires additional mechanisms to increase the DNA binding specificity of TCFs. For TCF family members containing a C-clamp, the presence of Helper sites near HMG sites is one such mechanism.

**Figure 8:**
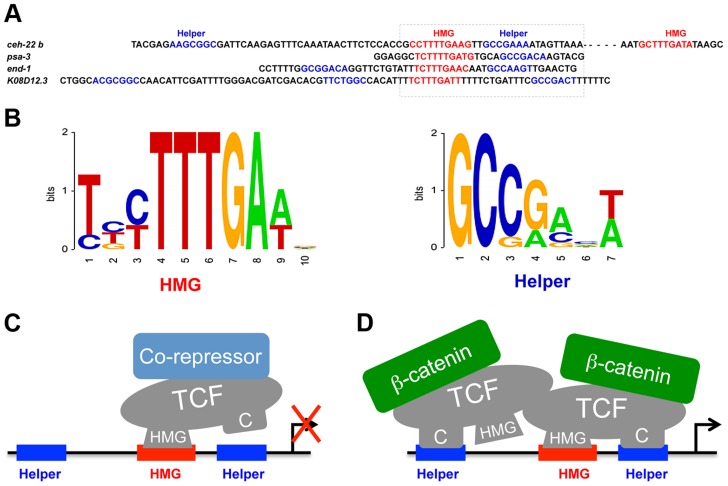
POP-1 consensus HMG and Helper sites and models for the TCF transcriptional switch. (A) Genomic sequences of the functional HMG and Helper sites, with the box indicating a HMG-Helper site pair with similar orientation in each WRE. (B) Sequence logos showing the consensus of HMG and Helper sites, based on the functional sites used in this study. (C, D) Model to explain the differential requirement of HMG and Helper sites in the TCF transcriptional switch, without (C) and with (D) Wnt/ß-catenin signaling . We propose that the DNA binding properties of TCF are influenced by co-regulators, with ß-catenin stabilizing the HMG-Helper site interaction. It is suggested that POP-1 may recognize HMG sites surrounded by two Helper sites as a dimer. This model does not preclude the existence of addition DNA-binding co-factors for POP-1 in either the absence or presence of signaling.

There are several similarities between the DNA binding sites for POP-1, TCF/Pan and vertebrate TCFs, but there are also some important differences. While the five functional HMG sites examined in this report have a consensus that is similar to other TCFs (Figure 8A) [11], [38], there are some differences, most notably HMG sites in the *ceh-22b* and *psa-3* WREs with CTTTTG instead of the traditional CTTTG (Figure 1A, 1B). POP-1 can bind to “classic” HMG sites used in POPTOP [77], but can also tolerate an extra T in the core of the HMG site. Whether this property is unique to POP-1 requires further study.

The functional Helper sites identified in this report are also similar but different from those in other systems. The *C. elegans* Helper consensus shown in Figure 7B, derived from the eight functional sites, is GCCRAnW (R = A/G; W = A/T). This is slightly different from the fly (GCCGCCR) and vertebrate (GCSGS) consensus sites [11]. The C-clamp domain of POP-1 is the most diverged of all the sequenced metazoan TCFs [9]. For example, the C-clamps of POP-1 and TCF/Pan are 59% identical/72% conserved, while TCF/Pan and human TCF4E are 83% identical/90% conserved. A more systematic comparison of Helper site binding from different TCF family members is required to determine if the above differences are due to intrinsic differences in their C-clamp domains.

Functional Helper sites are usually found near functional HMG sites in WREs [40], [41]. Mutation of individual HMG and Helper sites in fly WREs indicates that they act in pairs (Chang and Cadigan, unpublished observations) and this view is supported by studies of TCF binding in vitro, where the TCFs containing a C-clamp bind to adjacent HMG and Helper sites with high affinity [39]–[41]. However, the analysis of POP-1 binding clearly demonstrates that multiple Helper sites can augment binding of POP-1 to DNA containing a HMG site (Figure 3F; Figure S1A). An alignment of the four Helper-HMG clusters is shown in Figure 8A. All four WREs have a HMG-Helper pair in similar orientation, while three have additional Helper sites that contribute to *in vivo* WRE activity (Figure 2, 3 & 6). The mechanism by which POP-1 binds to multiple Helper sites and a single HMG site is not clear, though it seems likely that POP-1 homo-oligomerization is involved (Figure 8C; Bhambhani and Cadigan, unpublished results). While additional experiments are needed to resolve this issue, it seems clear that these results should be taken into account in designing computational searches for additional WREs.

To test whether knowledge of the importance of Helper sites can enhance our ability to detect WREs *in silico*, we conducted a genome-wide search for Helper-HMG site clusters. After secondary screening (see Results and Table S3) three candidates were tested for POP-1 binding (Figure 3F; Figure S2) and the one with strong binding (*K08D12.3*/*ZNF9*) was tested in a transgenic GFP reporter assay (Figure 3A). The reporter was active in several muscle cell types and the gut (Figure 3B), a pattern that has considerable overlap with a POP-1 transcriptional fusion [78]. Indeed, mutation of the HMG and Helper sites in the *K08D12.3* reporter caused a dramatic reduction in expression (Figure 3C, 3D). These results provide a proof of principle that novel POP-1 targets can be identified through HMG-Helper site directed computational searches.

There are some well-known targets of Wnt/ß-catenin signaling in *C. elegans* where WREs have not been identified, e.g., *mab-5* and *lin-39* [77], [79]–[82]. It is possible that these are indirect targets of the pathway, but an alternative is that the bona fide POP-1 binding sites have been missed because they contain sub-optimal HMG sites combined with multiple Helper sites. For example, one of the HMG-Helper site clusters identified in our computational search (among the initial 115 hits) is upstream of the *pha-4* gene [83], [84] (Figure S6A), whose expression is POP-1 dependent [85]. *egl-18* was also recently reported to be a direct target of POP-1, based on a partial reduction in reporter expression when a single HMG site was mutated [86]. Examination of the region near this site revealed another HMG-Helper site cluster nearby (Figure S7). While these observations require experimental validation, our work strongly suggests that the presence of Helper sites will in many cases facilitate the identification of functional POP-1 binding sites in Wnt targets.

### The TCF Transcriptional Switch

In this study, we have shown that four worm WREs require Helper sites for maximal expression (Figure 2; Figure 3; Figure 6). For one of these, plus a fly WRE where mutation of HMG sites resulted in substantial derepression of expression (Figure 6B; Figure 7E), Helper sites made little or no contribution to TCF basal repression (Figure 6C; Figure 7H). In addition, a TCF rescue assay in the developing fly wing demonstrated that the C-clamp was required for activation of Wnt readouts (Figure 7; Table 2) but was dispensable for repression of ectopic bristles (Figure 7L, 7L′; Table 2). Taken together, these results support a model where TCFs repress Wnt target gene expression in the absence of signaling through HMG domain-HMG site binding, but require HMG domain-HMG site and C-clamp-Helper site interactions to mediate activation (Figure 8C). Interestingly, a vertebrate TCF (TCF3) which is associated with repression of Wnt targets in the absence of signaling [87], [88] does not have a C-clamp, perhaps supporting the dispensability of C-clamp-Helper sites in basal repression.

It should be noted that a prior report has implicated the C-clamp as being required for the ability of POP-1 to repress endoderm genes in MS blastomeres and their descendants [23]. However, the deletion constructs used removed additional portions of POP-1 besides the C-clamp, and the precise region required for endoderm repression was not identified [23]. To resolve the discrepancy between this work and our results, more surgical mutations in the C-clamp should be employed to test its role in basal repression of Wnt targets.

One model to explain our data is depicted in Figure 8C. In this scenario, TCF DNA binding is allosterically regulated by associated co-regulators. Co-repressors would promote HMG domain-HMG site binding while ß-catenin would stimulate binding to both DNA motifs (Figure 8C). It is known that the C-clamp increases the affinity of TCFs for DNA containing HMG and Helper sites [39], [40], so this model predicts that TCFs would bind DNA more efficiently in the presence of high levels of nuclear ß-catenin. Indeed, in fly cells and tissues, Wg signaling causes a robust increase in TCF binding to WRE chromatin, as measured by Chromatin Immuno-Precipitation (ChIP) [89]–[92]. This increase in TCF binding is dependent on Armadillo, the fly ß-catenin [92]. This is in contrast to human LEF1, which lacks a C-clamp, where Wnt/ß-catenin signaling has no effect on the level of LEF-1 binding to a *c-myc* WRE [93]. Further biochemical analysis is required to determine the exact mechanisms involved in the differential requirement of Helper sites in the TCF transcriptional switch.

Transcriptional switches are a common mechanism for transcription factors (TFs) working in cell-cell signaling pathways [1]–[3]. There are hints that the DNA-binding properties of some of these TFs may be controlled by signaling in a manner analogous with TCFs. For example, the genome-wide distribution of retinoic acid receptor (RAR) is dramatically altered by ligand in differentiating mouse embryonic stem cells [94]. In another case, fly CSL (also known as Suppressor of Hairless), the TF that mediates Notch signaling, displays a transient increase in binding to Notch regulatory elements upon signal stimulation [95]. Interestingly, co-activator binding to mammalian CSL has been shown to stabilize binding to dimeric CSL binding sites [96]. Whether different binding sites contribute to the RAR or CSL transcriptional switches is not known. Given that fly and worm TCFs have two distinct DNA binding domains and DNA binding sites, their differential requirements in the transcriptional switch may be more readily discernable. But it is possible that other TFs operating as transcriptional switches have similar differential binding requirements in the absence or presence of signaling.

### Role of POP-1 in Post-embryonic Gut Physiology

While POP-1 is known to play important roles in gut development during embryogenesis [15], [18], [22], [73], its role in post-embryonic gut development is poorly understood. Our synthetic POPHHOP fluorescent reporter displays strong expression in ‘int9’ cells, the posterior most intestinal cells in the larval and adult stages (Figures 5A, 5G). This expression pattern is *pop-1* dependent (Figure 5H) and it has been reported that POP-1 is expressed in these cells during larval development [61]. Since these cells have been known to play an important role in the defecation behavior of *C. elegans* [56], we tested if POP-1 had any role in post-embryonic gut physiology and found that POP-1 regulates the frequency and regularity of the defecation cycle (Figures 5I, 5J, 5K).

The defecation behavior is comprised of rhythmic contractions of the posterior body wall muscles, followed by the anterior body wall muscle and eventually the enteric muscles expelling the fecal matter. Under conditions of constant feeding, these steps are highly regular [57] and are controlled by neuronal and non-neuronal signals [97]. The intestine is thought to be the pacemaker for this process [56], [58]–[60] with int9 cells exhibiting rhythmic calcium fluxes which initiate each cycle [56]. Hypomorphic *pop-1* mutants and animals with intestine-specific RNAi depletion of *pop-1* have a prolonged cycle and display significant arrhythmia (Figures 5I–5L). These data, along with the expression of POPHHOP in int9 cells suggests that POP-1 is required in these cells for pacemaker function. Since both the *pop-1(hu9)* [63], [64] and *pop-1(q645)* [50] alleles are point mutations in the ß-catenin binding domain, it is likely that POP-1 is working with Wnt/ß-catenin signaling to control this rhythmic behavior. The Wnt genes *egl-20*, *cwn-1* and *lin-44* are expressed close to or overlapping the int9 cells [98], [99] and SYS-1 is important for attachment of the posterior intestine to the rectum [18]. Hence they could be contributing to the non-neuronal pacemaker function of these posterior intestinal cells to regulate the pBoc step of the defecation cycle.

What are the downstream targets of Wnt/ß-catenin signaling in int9 cells? Based on our behavioral analysis, *pop-1* is in a class of mutants that show defecation cycle length and expulsion defects (Figure 5I-5L and Table 1). Of particular interest, loss of function mutations in a phospholipase Cβ (*egl-8*; Gene ID: 178537) lead to a dramatic increase in the cycle length with mutants displaying severe arrhythmia and expulsion defects [57], [59], [100]. *pop-1* mutants show a subtle phenotype compared to *egl-8*, which could be attributed to the hypomorphic alleles used in our study. Interestingly, egl-8 is expressed in the posterior intestine [100] and we found two HMG-Helper site clusters in the intronic regions of *egl-8* that could contribute to this pattern (Figure S6B). It will be interesting to see if the intestinal pattern of *egl-8* is dependent on POP-1 and whether the HMG-Helper sites we identified are functional.

There is clearly more work to be done in elucidating the role of Wnt/ß-catenin signaling in the *C. elegans* defecation cycle. For example, are the calcium fluxes originating from the posterior intestinal region [56], [58], [59] affected in *pop-1* mutants? While this and other questions remain, this report provides a powerful example of how knowledge of Helper sites working with HMG sites can not only lead to finding novel POP-1 targets, but also a better understanding of Wnt biology in *C.elegans*.

## Materials and Methods

### Plasmids

For *C. elegans* WRE reporters, specific mutations introduced into each HMG site and Helper site are listed in Supplemental Table S1. Site directed mutagenesis for all constructs was performed using the Quickchange II Kit (Stratagene). The WT sequences were replaced by subcloning the HMG or Helper mutant fragment into the PstI and BamHI site of *ceh-22b::VENUS* plasmid [42] (pJK1082, kindly provided by Dr. Judith Kimble), the PstI sites of *psa-3::GFP* plasmid [43] (kindly provided by Dr. Hitoshi Sawa) or the NaeI and AvrII sites of *end-1::GFP::H2B* plasmid [16] (kindly provided by Dr. Rueyling Lin). To generate the *K08D12.3::VENUS* plasmid, *ceh-22b* WRE was replaced by a 585 bp region spanning the TlSS and sequence upstream of the *K08D12.3* gene. PCR based cloning was used to insert this fragment at the SphI and SmaI site of the *ceh-22b::VENUS* plasmid. The fragment was amplified using the reverse primer 5′GCCAAT*CCCGGG*GATCCTTTCTGTCCGAGATTACTGCAA3′ with the start codon mutated (underlined) [48] and forward primer 5′TCGAA*GCATGC*CTGCAGCCGATTGCCGGAATGGCTTTGCGC3′. The *POPHHOP* plasmid was generated by cloning 6×HMG-Helper (6× GGAAGATCAAAGGGGGTAGCCGCCAGT) [40] upstream of a NLS-GFP in *pPD107.94* (also known as L3135) vector using the NheI sites. 6×Histagged full length POP-1 plasmid (*pRSETA-POP-1*) was kindly provided by Dr. David M. Eisenmann and the C-clamp mutant used in Figure 4 was generated from this plasmid. The *pxb-lacZ* plasmid was generated by subcloning the *pxb cluster3* WRE into the *pH-Pelican* vector as described previously [40] . *pUAS LEF1V5* and *pUAS LEF1C-clamp V5* were generated by PCR based cloning into *pUAST* vector. *pUAS LEF1-V5* was generated by subcloning a human *LEF1* fragment from an expression plasmid (kindly provided by Dr. Marian L. Waterman) into *pActin5.1* to introduce a V5 tag. PCR based amplification of *LEF1* was carried out using forward primer 5′CCCC*GGTACC*ATGCCCCAACTCTCCGGA3′ and reverse primer 5′CAGT*GAATTC*TGCGATGTAGGCAGCTGTCATTCTTGG3′ and inserted into the KpnI and EcoRI sites of the *pActin5.1* vector, with the stop codon mutated (underlined). *Lef1V5* was digested using the KpnI and PmeI sites and inserted into the KpnI and XbaI sites of *pUAST*. Prior to insertion, *pUAST* vector was restricted with XbaI and sticky ends filled in by Klenow to create blunt ends, followed by a restriction with KpnI. *pUAS-Lef1C-clamp V5* plasmid was generated by PCR based amplification of the C-clamp (i.e., KKCRARFGLDQQSQWCKPCRRKKKCIRYMEAL) from fly TCF/Pan and insertion into the EcoRI site of *pUAS Lef1V5* by non-directional cloning.

### *C. elegans* and *D. melanogaster* Transgenics and Genetics

Worm strains were derived from the wild-type *C.elegans* N2 Bristol strain and cultured using standard protocols. Transgenic strains with extrachromosomal arrays were generated by injecting WT or mutant versions of *ceh-22b::VENUS* (100 ng/µl), *psa-3::GFP* (50 ng/µl), *end-1::GFP::H2B* (100 ng/µl) or *K08D12.3::VENUS* (150 ng/µl) plasmid into N2 worms, along with coinjection marker *myo-2::RFP* plasmid (3 ng/µl) and *pActin5.1* (up to a total of 200 ng/µl). Stable integrants were generated by UV irradiation using a Stratalinker (Stratagene) at power 325. *POPHHOP* plasmid (1 ng/µl) was injected along with a *dpy-20(+)* plasmid (50 ng/µl) and *pBluescript* (100 ng/µl) and stable integrants generated by gamma-irradiation. Animals with integrated transgenes were outcrossed at least three times. Transgenic *POPTOP* (*7× HMG::mCherry*) strain was kindly provided by Dr. Paul W. Sternberg [55]. It should be noted that the proximal promoter and 3′ UTR of POPHHOP and the POPTOP constructs are different, which could account for some of the differences in expression of these two synthetic reporters. *POPHHOP* was crossed into a *pop1(hu9)* [64] background and analyzed at different stages. All reporter strains were maintained at 25°C except for *psa-3::GFP* transgenic analysis during which synchronized L1s were grown at 20°C for 9 hours only for the reporter analysis. *pop-1(q645)* strain (JK2944) was obtained from *Caenorhabatitis* Genetics Center (CGC).

Transgenic *pxb-lacz*, *Lef1* and *Lef1 C-clamp* flies were generated by P-element transgenesis (performed by BestGene Inc.). *w1118* was obtained from Bloomington Stock Center. *C96::Gal4* was kindly provided by Dr. Rolf Bodmer [71]. The *TCF/Pan RNAi* line (#25940) was obtained from Vienna *Drosophila* RNAi Center. All fly crosses were performed at 25°C.

### Imaging

Methods for mounting and viewing *C.elegans* larvae and embryos by Nomarski optics have been described previously [101], [102]. *ceh-22b::VENUS*, *psa-3::GFP*, *end-1::GFP::H2B*, *K08D12.3::VENUS* and *myo-2::RFP* reporter expression was analyzed by fluorescence on a Olympus BX61 motorized X-drive microscope. Images were taken using a Hamamatsu ORGA-ERCA-CCD camera, with a specific exposure time for each WRE reporter and multiple focal planes were merged to obtain the representative image. Deconvolution was performed using slidebook 5.0 software and the nearest neighbors method. *POPHHOP* and *POPTOP* reporter expression was analyzed using a Zeiss Axioscope microscope equipped with a Zeiss Axiocam digital camera. *pxb-lacZ* WRE images were obtained using a Leica triple channel confocal microscope DM6000B-CS and multiple focal planes were merged to obtain the representative image. All images were processed using Adobe Photoshop 8.0.

### EMSA

Full-length 6×His-POP-1 was expressed in *E.Coli* and purified using nickel beads (Sigma). ESMA was performed as described previously [40] using 6% native gels. POP-1 (300–900 ng unless otherwise indicated) in 10% glycerol and the biotin labeled DNA probes (4–8 femtomoles unless otherwise indicated) were incubated with 50 ug/ml poly (dI-dC), 0.05% NP-40, 5 mMMgCl2 and 2 µl of 50% glycerol in the presence of binding buffer (10 mM Tris–HCl, pH 7.5, 50 mM KCl, 1 mM DTT) in a final volume of 20 µl for 5 min on ice and 25 min at room temperature. For the competition assays, unlabeled probes were incubated with the reaction mixture containing POP-1 for 10 min prior to adding the labeled probe.

### Fixation, Immunostains and Immunoblots

For *ceh-22b::VENUS* fluorescence analysis, worm larvae were fixed in 4% paraformaldehyde (in M9) for 15 min, using a protocol adapted from the whole-mount freeze-cracking method [103], without any immunostaining. For *pxb-lacZ* WRE analysis, fly embryos were fixed and immunostained using anti-lacZ and anti-Wg as described previously [40]. For comparing Lef1 and Lef1 C-clamp expression, wing imaginal discs from third instar larvae were dissected, immediately suspended in boiling 5×-Laemmli buffer, homogenized and boiled for 5 min. Immunoblots were performed using anti-V5 and anti-tubulin as described previously [104].

### Behavioral Analysis

N2 worms and *pop-1* mutants were maintained at 20°C, and scored for defecation at room temperature (∼23°C) using a Leica MZ16 F stereoscope at a 115× magnification. The protocol was adapted from a previous report [57]. Briefly, ten larvae (from synchronized L1s grown at 20°C for 15 hours) were transferred to a seeded NGM plate each time and allowed to settle down at 20°C for one hour followed by at least twenty minutes at room temp before assaying them for defecation at early L2. Pharyngeal pumping was observed for at least one minute in each worm before starting the defecation assay, to ensure the overall health of the animal and to confirm that they were not in the L1/L2 lethargus stage. Defecation cycle length was defined as the time between two consecutive pBocs [105]. Each larva was scored for eight consecutive cycles (nine consecutive pBocs) and the mean was calculated. The mean cycle lengths were used to calculate the mean, median and standard deviations for each genotype. Expulsion was observed at ∼three seconds after a pBoc. *pop-1(hu9)* worms were maintained as a homozygotes [64] and scored for defecation. The *pop-1(q645)* (JK2944; CGC) strain segregates as WT- green-fluorescing heterozygotes, and non-fluorescing homozygotes. *pop-1(q645)* hermaphrodites are sterile due to severe gonadal development defects [50]. Non-fluorescing *pop-1(q645)* larvae were scored for defecation cycles, transferred to individual plates and allowed to grow to adulthood to confirm that they were sterile and had a protruding vulva [50]. Four homozygotes for each allele were genotyped to confirm the point mutations for *pop-1(hu9)* (G to A)(E47 to K) [64] and *pop-1(q645)* (T to A)(D9 to E) [50] in the N-terminal β-catenin binding domain using forward primer 5′CTCCATGGCCTAACTTCCGCGGACC3′ and reverse primer 5′GTCGAAAGGCAATTGAGGTGGTCC 3′.

RNAi feeding of N2 and OLB11 strains were performed as described [106] using the pop-1 dsRNA expressing strain from the Ahringer RNAi library [51].

### Wing Mounting

Adult flies were stored in 70% ethanol and soaked in 100% ethanol for two hours before dissection in 100% ethanol. Dissected wings were transferred into Xylene (Fisher) and mounted on a slide with Permount (Fisher). Wing notch and bristle images were obtained using the Nikon Eclipse 800 microscope. All images were processed using Adobe Photoshop 8.0.

## Supporting Information

Figure S1 Helper sites are important for binding of POP-1 to *ceh-22b* and *psa-3* WRE probes. EMSA analysis of a 69 bp *ceh-22b* WRE probe (A) and 47 bp *psa-3* WRE probe (B) both of which show a POP-1 dependent shift (lane 2). For *ceh-22b*, an excess of unlabeled wildtype (WT) oligonucleotide competes with the labeled probe for POP-1 binding (lanes 3 & 4). Much less competition is observed when oligonucleotides containing mutations in the Helper 1 (lanes 5 & 6) or Helper 2 (lanes, 7 & 8) sites are used. No competition is observed with DNA containing mutations in both Helper sites (lanes 9 & 10) or the HMG2 site (lanes 11 & 12). For the *psa-3* probe, efficient competition is observed with excess of unlabeled WT DNA (lanes 3–5), which is greatly reduced with Helper mutant (lanes 6–8) or HMG mutant (lanes 9–11) DNA. Black arrowheads represent the DNA-protein complex and white arrowheads represent unbound probe. ‘*’ represents a band which was seen in some experiments which we suspect is a POP-1 degradation product.
(PDF)

Figure S2 Secondary screen for putative WREs using EMSA reveals strong binding of POP-1 to a HMG-Helper site cluster from the *K08D12.3* locus. (A) Probes derived from genomic sequences of 3 genes identified in a genome-wide search for one HMG site - two Helper sites clusters. (B) The long probe derived from a region upstream of the *K08D12.3* gene showed robust binding to POP-1 (lanes 1–3). A shorter probe lacking the first Helper site has weaker binding (lanes 4–6). Two other clusters were negative for binding to POP-1 (lanes 7–12). A *ceh-22b* probe was used as a positive control (lane 13). Black arrowheads represent the DNA-protein complex and white arrowheads represent unbound probe.
(PDF)

Figure S3 Helper sites are important for binding of wild-type and C-clamp mutant POP-1 to *ceh-22b* WRE probes. EMSA analysis of the *ceh-22b* WRE probe containing two Helper sites (lanes 1–5) and a probe where these motifs are mutated (lanes 6–10). Recombinant wild-type or C-clamp mutant POP-1 (400 & 800 ng/reaction) was added where indicated. Either probe was used at 1.5 femtomoles/reaction. A dramatic reduction in binding was observed in the C-clamp mutants (lanes 4, 5, 9 &10) or when wild-type POP-1 was incubated with the Helper site mutant probe (lanes 7 & 8).
(PDF)

Figure S4 Expression pattern of the *POPHHOP* reporter construct. (A–F) DIC images with GFP fluorescence overlay. The reporter is expressed with a high penetrance during the L1 stage, in the int9 cells, the tail neurons and VC neurons (A) and during L3 stage in DTCs (B). During the L1 stage, expression was occasionally seen in muscle cells (C), seam cells (D–G) and QL daughters (E–F). Scale bar = 10 µm.
(PDF)

Figure S5 Expression of human Lef1 and Lef1-C-clamp chimera in wing imaginal discs. (A) Cartoon of human LEF1 and LEF1-C-clamp fusion showing the β-catenin binding domain (green), the HMG domain (red), the basic tail (orange), the C-clamp (blue), a linker (gray) and the V5 epitope (hatched box). Immunoblot showing the expression levels in dissected wing discs from two lines (A and B) of V5 tagged Lef1 (*) or the Lef1-C-clamp chimera (**).
(PDF)

Figure S6 HMG-Helper site clusters in putative POP-1 targets. A) Genomic sequence of a region upstream of the *pha-4* TlSS showing the putative HMG (red) and Helper sites (blue). B) Genomic sequence of *egl-8* showing clusters i and ii with HMG (red) and Helper (blue) sites identified in the intronic regions of *egl-8*. The flanking exon sequences are highlighted in green.
(PDF)

Figure S7 Putative HMG and Helper sites in a known Wnt target. Genomic sequence of a region upstream of the TlSS of *egl-18*, which was used for a transcriptional fusion reporter [86]. The first HMG site (red) was shown by Gorrepati et al to be functional [86]. In addition, there is another predicted HMG site and several Helper sites (blue) further downstream.
(PDF)

Table S1 Mutations introduced in the HMG or Helper sites for reporter constructs and EMSA experiments. For each motif the wild-type sequence is shown in the first row with mutant substitutions (lower case) in the second row for either reporter gene constructs (mutT) and/or EMSAs (mutE).
(DOCX)

Table S2 Score Matrices used for HMG and Helper computational search using target explorer [45].
(DOCX)

Table S3 List of 19 hits from a genome-wide search for HMG-Helper site clusters. Hits contained at least two Helper and one HMG site within 50 bp. The cutoffs for Helper and HMG sites were 5.51 and 6.69, using the weighed matrices shown in Table S2. The sequences shown could be in either forward or reverse orientation. The position corresponds to the 500 bp upstream of each gene that was searched; position 1 corresponds to −500 (from the first codon) onward toward position 500 (−1 from first codon).
(DOCX)
