# Supplementary material for: Distinct DNA Binding Sites Contribute to the TCF Transcriptional Switch in C. elegans and Drosophila
Source: PLoS Genet. 2014 Feb 6;10(2):e1004133. doi: 10.1371/journal.pgen.1004133 (PMC3916239; doi:10.1371/journal.pgen.1004133)
Supplement: Figure S1 — Helper sites are important for binding of POP-1 to ceh-22b and psa-3 WRE probes. EMSA analysis of a 69 bp ceh-22b WRE probe (A) and 47 bp psa-3 WRE probe (B) both of which show a POP-1 dependent shift (lane 2). For ceh-22b, an excess of unlabeled wildtype (WT) oligonucleotide competes with the labeled probe for POP-1 binding (lanes 3 & 4). Much less competition is observed when oligonucleotides containing mutations in the Helper 1 (lanes 5 & 6) or Helper 2 (lanes, 7 & 8) sites are used. No competition is observed with DNA containing mutations in both Helper sites (lanes 9 & 10) or the HMG2 site (lanes 11 & 12). For the psa-3 probe, efficient competition is observed with excess of unlabeled WT DNA (lanes 3–5), which is greatly reduced with Helper mutant (lanes 6–8) or HMG mutant (lanes 9–11) DNA. Black arrowheads represent the DNA-protein complex and white arrowheads represent unbound probe. ‘*’ represents a band which was seen in some experiments which we suspect is a POP-1 degradation product. (PDF) [file pgen.1004133.s001.pdf]

A

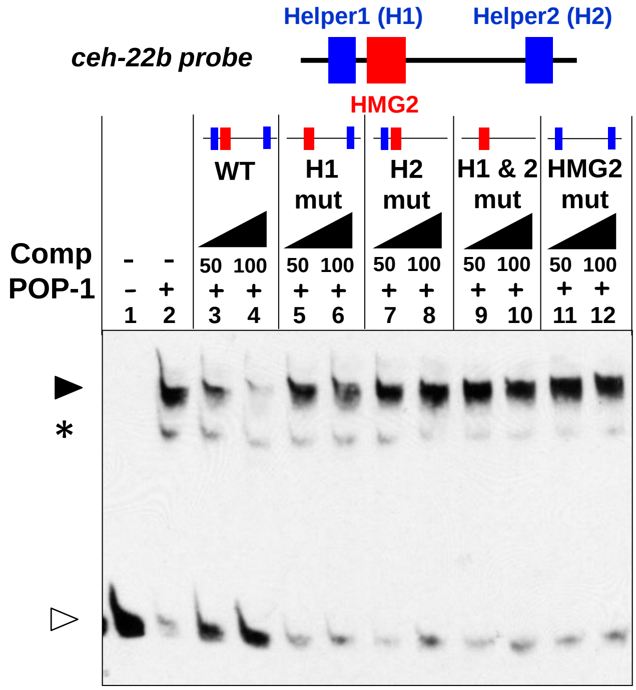

ceh-22b probe TACGAGAAGCGGCGATTCAAGAGTTTCAAATAACTTCTCCACCGCCTTTTGAAGTTGCCGAAAATAGTT

Helper2 HMG2 Helper1

B

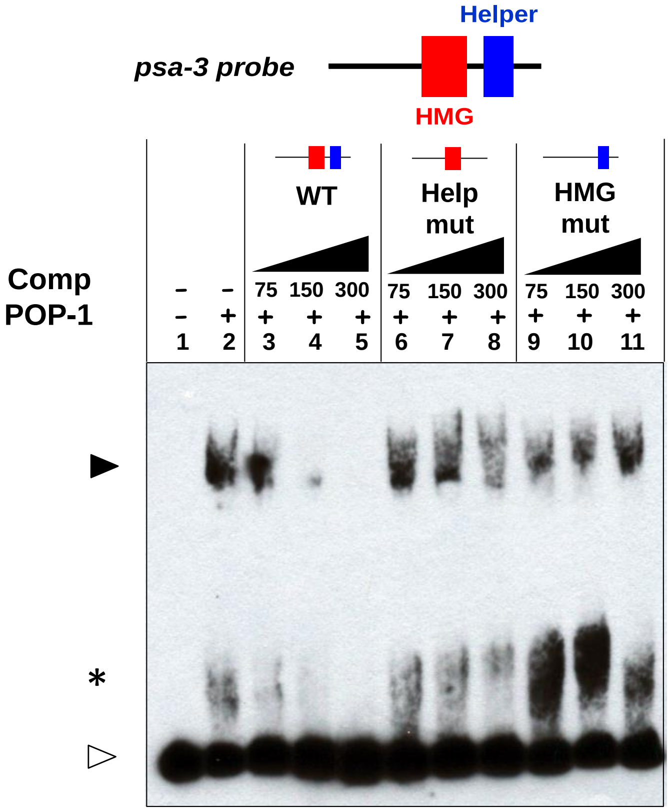

psa-3 probe ACCGTTATGGCCGTGGAGGCTCTTTTGATGTGCAGCCGACAAGTACG

HMG Helper
