# Supplementary material for: Distinct DNA Binding Sites Contribute to the TCF Transcriptional Switch in C. elegans and Drosophila
Source: PLoS Genet. 2014 Feb 6;10(2):e1004133. doi: 10.1371/journal.pgen.1004133 (PMC3916239; doi:10.1371/journal.pgen.1004133)

A

	Helper		Helper	HMG	Helper

K08D12.3

long CTGGCACGCGGCCAACATTCGATTTTGGGACGATCGACACGTTCTGGCCACATTTTCTTTGATTTTTTCTGATTTCGCCGACTTTTTTC

short ACACGTTCTGGCCACATTTTCTTTGATTTTTTCTGATTTCGCCGACTTTTTTC

Y66D12A.9 TTGGCGCCGAGCACATATTTCCCATCGGAAGCTTTGAAATAGTCGAGCCCGCGGCCACTT

R05G6.5 CGGTCGCCGCGCCAATGTGCTTGCCTTTGAACTCTCAATTTGCTGTTGTGGCGTGAT

ceh-22 b TACGAGAAGCGGCGATTCAAGAGTTTCAAATAACTTCTCCACCGCCTTTTGAAGTTGCCGAAAATAGTTAAA

B

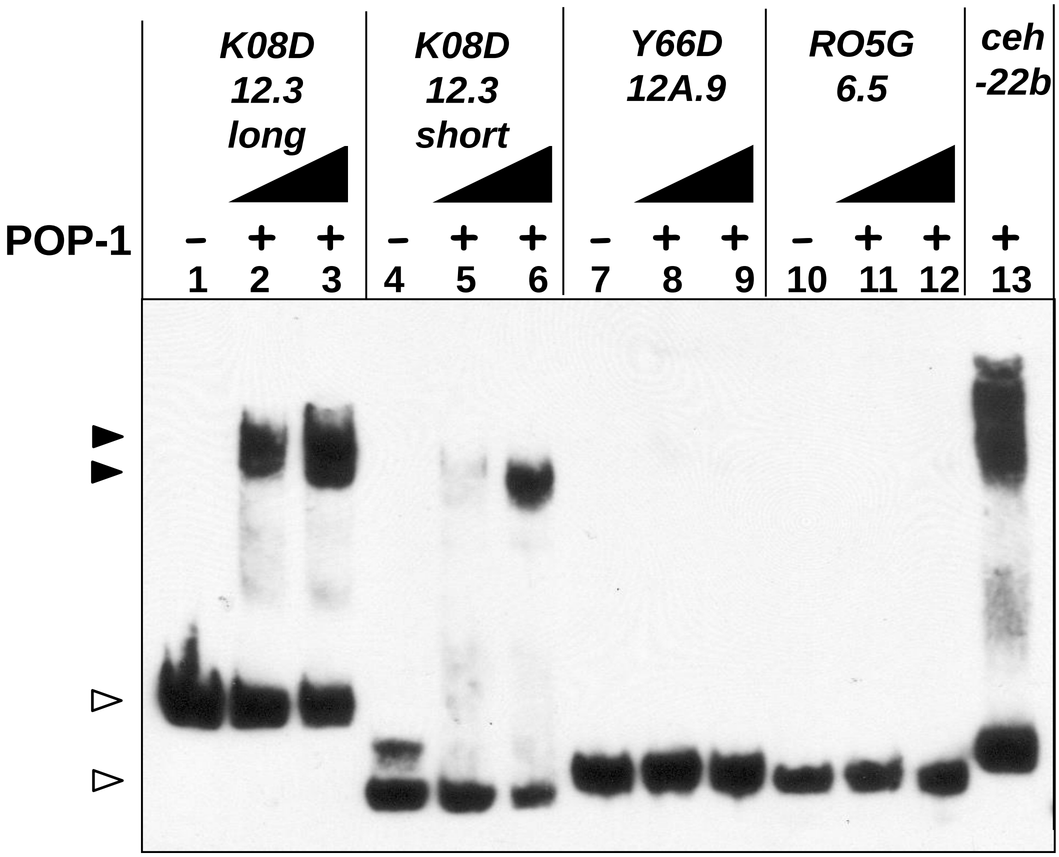

Supplement: Figure S2 — Secondary screen for putative WREs using EMSA reveals strong binding of POP-1 to a HMG-Helper site cluster from the K08D12.3 locus. (A) Probes derived from genomic sequences of 3 genes identified in a genome-wide search for one HMG site - two Helper sites clusters. (B) The long probe derived from a region upstream of the K08D12.3 gene showed robust binding to POP-1 (lanes 1–3). A shorter probe lacking the first Helper site has weaker binding (lanes 4–6). Two other clusters were negative for binding to POP-1 (lanes 7–12). A ceh-22b probe was used as a positive control (lane 13). Black arrowheads represent the DNA-protein complex and white arrowheads represent unbound probe. (PDF) [file pgen.1004133.s002.pdf]
