# Supplementary figures and images for: Distinct DNA Binding Sites Contribute to the TCF Transcriptional Switch in C. elegans and Drosophila
Source: PLoS Genet. 2014 Feb 6;10(2):e1004133. doi: 10.1371/journal.pgen.1004133 (PMC3916239; doi:10.1371/journal.pgen.1004133)

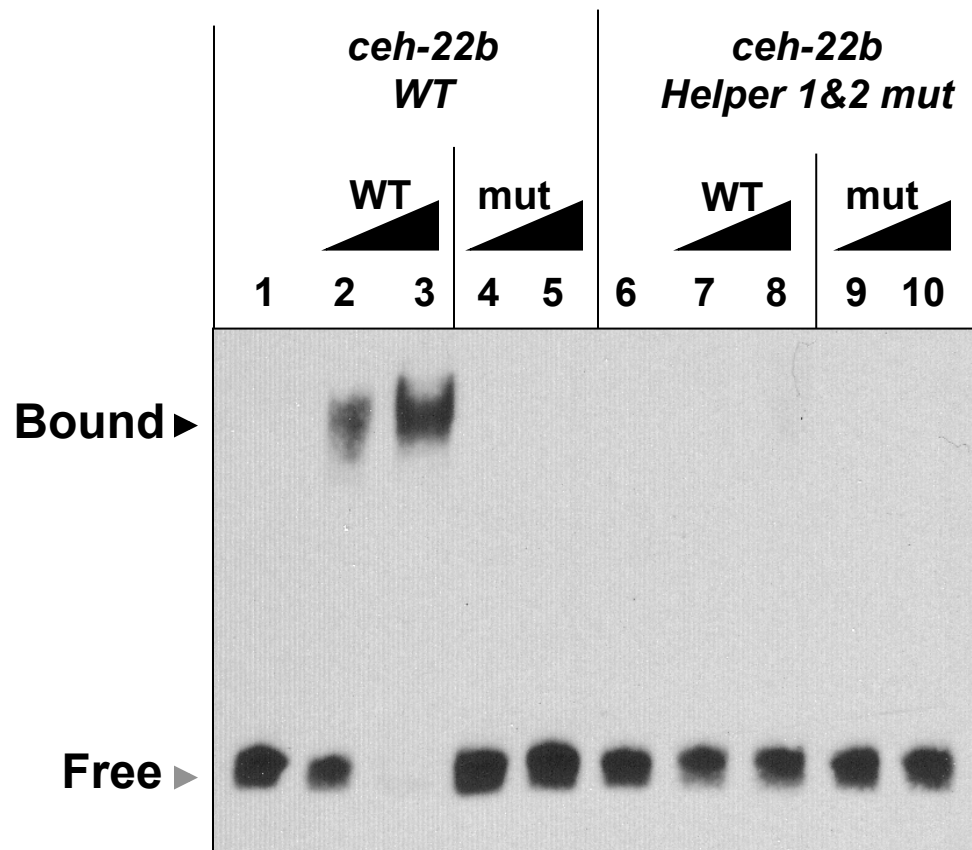

Supplement: Figure S3 — Helper sites are important for binding of wild-type and C-clamp mutant POP-1 to ceh-22b WRE probes. EMSA analysis of the ceh-22b WRE probe containing two Helper sites (lanes 1–5) and a probe where these motifs are mutated (lanes 6–10). Recombinant wild-type or C-clamp mutant POP-1 (400 & 800 ng/reaction) was added where indicated. Either probe was used at 1.5 femtomoles/reaction. A dramatic reduction in binding was observed in the C-clamp mutants (lanes 4, 5, 9 &10) or when wild-type POP-1 was incubated with the Helper site mutant probe (lanes 7 & 8). (PDF) [file pgen.1004133.s003.pdf]

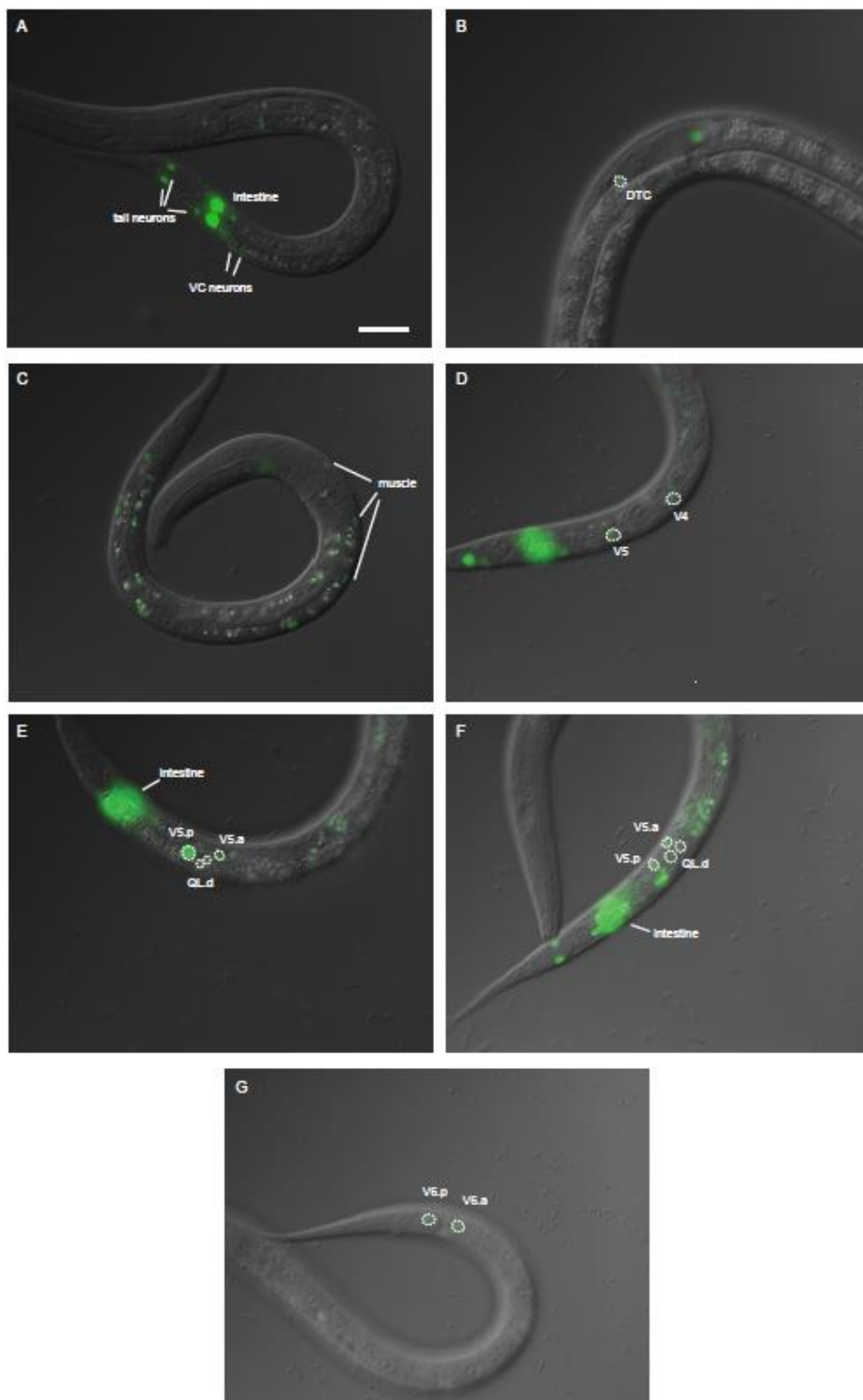

Supplement: Figure S4 — Expression pattern of the POPHHOP reporter construct. (A–F) DIC images with GFP fluorescence overlay. The reporter is expressed with a high penetrance during the L1 stage, in the int9 cells, the tail neurons and VC neurons (A) and during L3 stage in DTCs (B). During the L1 stage, expression was occasionally seen in muscle cells (C), seam cells (D–G) and QL daughters (E–F). Scale bar = 10 µm. (PDF) [file pgen.1004133.s004.pdf]

**A**

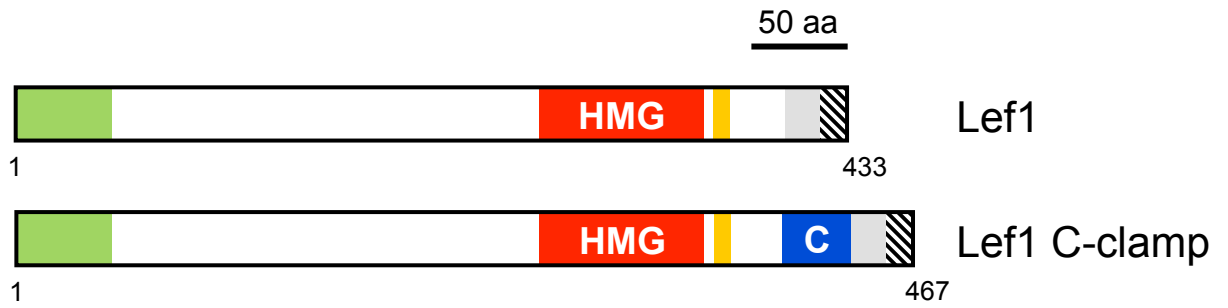

**B**

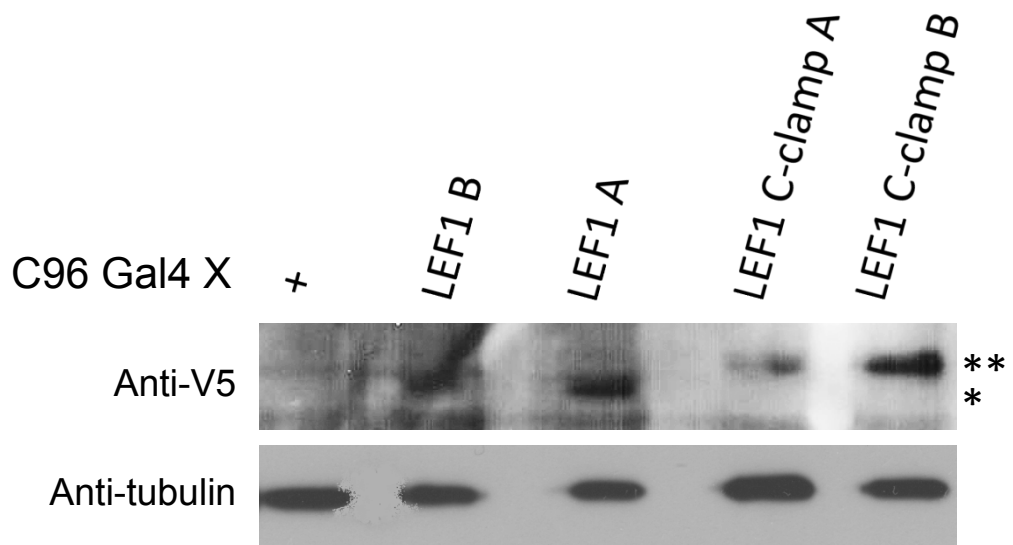

Supplement: Figure S5 — Expression of human Lef1 and Lef1-C-clamp chimera in wing imaginal discs. (A) Cartoon of human LEF1 and LEF1-C-clamp fusion showing the β-catenin binding domain (green), the HMG domain (red), the basic tail (orange), the C-clamp (blue), a linker (gray) and the V5 epitope (hatched box). Immunoblot showing the expression levels in dissected wing discs from two lines (A and B) of V5 tagged Lef1 (*) or the Lef1-C-clamp chimera (**). (PDF) [file pgen.1004133.s005.pdf]
