# Supplementary material for: Distinct DNA Binding Sites Contribute to the TCF Transcriptional Switch in C. elegans and Drosophila
Source: PLoS Genet. 2014 Feb 6;10(2):e1004133. doi: 10.1371/journal.pgen.1004133 (PMC3916239; doi:10.1371/journal.pgen.1004133)
Supplement: Figure S6 — HMG-Helper site clusters in putative POP-1 targets. A) Genomic sequence of a region upstream of the pha-4 TlSS showing the putative HMG (red) and Helper sites (blue). B) Genomic sequence of egl-8 showing clusters i and ii with HMG (red) and Helper (blue) sites identified in the intronic regions of egl-8. The flanking exon sequences are highlighted in green. (PDF) [file pgen.1004133.s006.pdf]

A ***pha-4***

ctctcatttcgtcatcgttcttcagctcttgaaattgccgtttgtatcatttggtgctatat
attgcattttaagagatttccttgccgcttttcctctcatcaacccttaagctttcgccga
aattgcccccggacatattttcaatttcttcacatctgaatcctcctccctcctctatccca
cttgtatgtaattctataccacaccacctcattcttagtatcctagtacgtgctaggctagg
acacaaaattgggtcaagtggaaaagagatattttggaaaattcaatgaaaaatttgagaac
tttttaacaatgaaaaaaattaaactattttgtttgaaggtcatcaaagaggagccagagtc
ggaacctgactctgaagccgagccagccacaaccacgaattcaacggattcagaagattctg
tggaacaggaaaataaaaagttattggaaaccgagaagaatcgaaaacgcgagcagaaacat
aaa

B ***egl-8***

i)

gttcaagagctgtttgttaaattgtgagtttttcttctcaaaaataaaattactgacttt
tttgttgtaattgcagatttctgatatattggaaaatgtatttttcaaaaaaaaattgcc
ttaactaacagaaaatgtactattacaccgattattagtacttataattttttttttgaa
agttggaaaatatttaaagtttgtaaaaattctataaataatattgaatattataacgg
taaaatataatatttatagaatttttcaattttcaaaaaagtactgtttgtcatttgtt
tttgtttgttagaatacctttaatagcggtaccgaaatctgggaaatattttcaaatga
ctccaaattttcgcctgattccgaatatctatgtgaaaaaaagtgaaaaaaaacatccct
tattttatatttcatttccaattcccaatttcatttgtgcccgcattactttttcaaac
gcgcgcccaaataaatttcattagagcgcgtttgctttgtgtcgatttacgggagctct
ccatttacaaaaaaaattaacgctttttttaaacagtttttaagcgagtttcctactttt
ttaatcgatttcagcggaacggtttttcttgaaatatcgtttttaaggtcaattgatatg
ataataaaatatattgttgaccttaaaacacattaaaaaaacttcgacaagaaccgacaa
aaagaagaaaaccggttgaaaacgataaagataaaacatttcgtaaatatacacaaatct
cgtaaatcgacacatggcgttttggcgcgaaaattcggcgtttgaaaaacttttcaaaag
tttcactgtcttatttggtaattatgtcacccactggcgctgctccacctttaaaaata
ttttcctcttgcagatccggacagaaagagtacctaaccaaagagcgactcatcaatttt

ii)

tggctgtggataaatttaaggttagttagtgggtggcagaaacagatagcagccgatatt
atgttagtcctgcaatatatcggctgctgtttcaaagaaggagtcgccatttttcgggtt
ggtcctgttatgtgtcgtttgctacattgaagtacttcaaaagagcagcggacactatgc
gggttagtcctggaacatgtcgactgtcaattagaagtggcagctgacatcttacgggag
agtcctgtgatgagtcggctgccaatttgtgtggcagcggacattatgggcctttgtttt
ctgctactccaaaaatcgttaacaaagttctgcaatacgtcggctgccatttaaaagtgg
cagctgacatttaacgagttagtccagcaatctgacgacagcgatttcgatgtagcagcc
gacatattgtgggttaattcggcaatgtgtcgggtgccatttcatagtggcagctgacat
ttcacgggtcagttcttcgatttatcgaatattactatcaagtagtggccggcattttg
ctgctattcctaaaaatgcgtaaactagggtcttgcaatatgtcggctgccaaatcaaag
tagcagccgaccatccgcgttttcactacaaaaacatatcaaaacttttctctaagaccc
tggccaaactgactcgaacatttactcgaaagtcggcgtccgcttcgccagcccacccaa
aaatcgggatcggtcacccaccggctggctcagctccgaaaccttcaacatcagtcgggg
cacccgaacggcttcaggttaccccacctagaactccctctttctcaacttttttttca
aatgaatatgatcctcctgcaagtctcgtgaatgatcttatagatatccagtttttaaa
ctttccaatttcttgtgattttaaaaaccgagtaaatttttcaggttgacccaatcgaag
