# Supplementary material for: Distinct DNA Binding Sites Contribute to the TCF Transcriptional Switch in C. elegans and Drosophila
Source: PLoS Genet. 2014 Feb 6;10(2):e1004133. doi: 10.1371/journal.pgen.1004133 (PMC3916239; doi:10.1371/journal.pgen.1004133)
Supplement: Figure S7 — Putative HMG and Helper sites in a known Wnt target. Genomic sequence of a region upstream of the TlSS of egl-18, which was used for a transcriptional fusion reporter [86]. The first HMG site (red) was shown by Gorrepati et al to be functional [86]. In addition, there is another predicted HMG site and several Helper sites (blue) further downstream. (PDF) [file pgen.1004133.s007.pdf]

egl-18

gcccaaataaaggaaggatgttgaggaagaagttgaggcaaaaagagagagacttcgtttcgaaga
gaagatcgcaatcaactcttctgatcagcagccatccaatcgaatcgataagaatgtttg
aagattaccatcttctcactaacatcgtcacacacacacacacatacactcacattctcc
actgcattcctttgttcggactcatcacggactcatcacctcatcgtttcttgtgttagg
acaccactgtttacgggctcttagaccggttatggtgcatcgtctccttgtcagatggac
caccagggtgttaccgccacacaggttctcggcaggtgttatctgagattagctgagaat
tcagccttgtcctggaaaattgtttgatttttttacctggcatattagctcagggctcggt
gaaagctttttcacagatttctccgctcgattcgaccaattttgagcatttataccatgg
gtggccggcacttggcgttttgccaatttttttgtcggtaagttgccggtttgccgagtt
gccggaaaacttcaatttcggcaatttgccgattttccggaagttgcaattttttgccaaa
aaaattccgatttgccgtttgccgtacataagatttgccggaaatcatagaatttgtttactt
ttcaaaaggatgcgtacacttttttgccgattaaaattggaattcagaatttaaaaaa
aaacgtgcaaattcccaatttgcagaaaatgttcggcaaatcggcaaaccggcacattgt
cgatttgccggaaatttcaattccggcagtttgccgatgtgccgcccatccttaatctat
accgaaactcaaattttcaaaaaaaaaattttttttttaaattgataaaaactattgtag
caaatcaaaaaaatgtttaaaaaaacatggcaaagattccaattttgtttttggaaattt
tgtaataaggtgttttagagactttcaaaacgccatcactttgctgtacctgaaccgggg
cagctgaatttttcggagggatcattattagcactttttttatcgaaaattacaacggtt
ttccggcaaaatctgtttttttccagcttttttatgcaaaaaaaaagacaccaattttga
aaaattgatatatttcgagtttccttgacacttggcacagttccaaacaacttccgttat
cgacaagctaaaatttggcacatctacaattatcgattaggtttagtgtcggggtgatac
tacgtgcataatttgtcgtacaacaacacaacaaatcgtctcccctcttccccccccccc
ccccccctatccacctaatttccacgtttgtccggctgggggaaaggggcggagacggg
cggtgagtagcgttcttctccacacaatatcccgcccatttttcttcgcgttgatcagtgt
cattttatggttatttacttgttggtccattcttgtcagtaaggagggcctcccttttta
aagttttgacctatatttttgattattttttgtctagtttcagcttgaactaagccgatt
ttaggccgaaatagtccgcatttttctgattcatagcttaaatttaatctccaaattgct
aattttgagtagatccctatgtttatgctacgtatttttagccccaaatagtttttaaat
aggcttttattgataaaaattgaccctttaagaattaaaactaagctatttatcggcctg
aattctccatacaaaacggctattttaggccgtttaggccgtcaatttctggtacctcca
tagtagtacattttaaggttcaaataaaattgtcccccgaaaaatcagttttttagtcca
aaatcacccattttaagccctaccccaactctgttcaggtcaaaatttttatcctcgtact
ctcttgatatttacttcccctgcacattttccctctaaattttccatttttttgcagtgt
ctccacacagtctat
