# Supplementary material for: Distinct DNA Binding Sites Contribute to the TCF Transcriptional Switch in C. elegans and Drosophila
Source: PLoS Genet. 2014 Feb 6;10(2):e1004133. doi: 10.1371/journal.pgen.1004133 (PMC3916239; doi:10.1371/journal.pgen.1004133)
Supplement: Table S1 — Mutations introduced in the HMG or Helper sites for reporter constructs and EMSA experiments. For each motif the wild-type sequence is shown in the first row with mutant substitutions (lower case) in the second row for either reporter gene constructs (mutT) and/or EMSAs (mutE). (DOCX) [file pgen.1004133.s008.docx]

| **WRE** | **Site** | **Type** | **Sequence** | **Reference** |
| --- | --- | --- | --- | --- |
|  |  |  |  |  |
| *ceh-22b* | HMG1 | WT  mutT & mutE | AATGCTTTGATATA  AATGCgggGATATA | [[42](#_ENREF_42)] |
|  | HMG2 | WT  mutT & mutE | CGCCTTTTGAAGTT  CGCCggggGccGTT |  |
|  | Helper1 | WT  mutT & mutE | ATCGCCGCTTCTC  ATCtaataggCTC | This report |
|  | Helper2 | WT  mutT & mutE | GTTGCCGAAAATA  GTTtaatgggATA |  |
|  | | | | |
| *psa-3* | HMG | WT  mutT & mutE | GCTCTTTTGATGTG  GCTCTggaGATGTG | [[43](#_ENREF_43)] |
|  | Helper | WT  mutT  mute | GCAGCCGACAAGT  GCAtaatACAAGT  GCAtaatgagAGT | This report |
|  | | | | |
| *end-1* | HMG | WT  mutt | ATTTCTTTGAACAA  ATTTCcccGAACAA | [[16](#_ENREF_16)] |
|  | Helper1* | WT  mutt | TTGGCGGACAGGT  TTttattACAGGT | This report |
|  | Helper2 | WT  mutt | AATGCCAAGTTGA  AATtaacAGTTGA |  |
|  | | | | |
| *K08D12.3* | HMG | WT  mutT & mutE | TTTTCTTTGATTTT  TTTTCTgTGcgTTT | This report |
|  | Helper1 | WT  mutT  mutE | TTGGCCGCGTGCC  TTGtaatCGTGCC  TTGtaatatgGCC |  |
|  | Helper2 | WT  mutT  mutE | GAGGCCAGAACGA  GAGtaacGAACGA  GAGtaactccCGA |  |
|  | Helper3 | WT  mutT  mutE | TTCGCCGACTTTT  TTCtaatACTTTT  TTCtaatcagTTTT |  |
|  | | | | |
| *pxb* | HMG1 | WT  mutT | AAGCGTTTGTACAG  AAGCGTgTGgcCAG | [[40](#_ENREF_40)] |
|  | HMG2 | WT  mutT | AACGCTTTGAAGCC  AACGCTgTGccGCC |  |
|  | Helper1 | WT  mutT | TGTGCCGCCCGAT  TGTtCaGCCCGAT | This report |
|  | Helper2 | WT  mutT | GAAGCCGCCTCCC  GAAtCaGCCTCCC |  |
| *Motif could be in either orientation | | | | |
