# Supplementary material for: Distinct DNA Binding Sites Contribute to the TCF Transcriptional Switch in C. elegans and Drosophila
Source: PLoS Genet. 2014 Feb 6;10(2):e1004133. doi: 10.1371/journal.pgen.1004133 (PMC3916239; doi:10.1371/journal.pgen.1004133)
Supplement: Table S2 — Score Matrices used for HMG and Helper computational search using target explorer [45]. (DOCX) [file pgen.1004133.s009.docx]

Score matrix of HMG sites:

| A | -1.79 | -1.79 | -1.79 | -1.79 | -1.79 | -1.79 | 1.08 | 0.25 |
| --- | --- | --- | --- | --- | --- | --- | --- | --- |
| C | 0.98 | 0.98 | -1.79 | -1.79 | -1.79 | -1.79 | -1.79 | -1.79 |
| G | -0.00 | -1.79 | -1.79 | -1.79 | -1.79 | 1.47 | -1.79 | -1.79 |
| T | -0.33 | 0.25 | 1.08 | 1.08 | 1.08 | -1.79 | -1.79 | 0.61 |

Alignment matrix of HMG sites:

| *ceh-22* HMG1 | : | GCTTTGAT | **score=7.38** |
| --- | --- | --- | --- |
| *ceh-22* HMG2 | : | CTTTTGAA | **score=7.27** |
| *psa-3* HMG | : | CTTTTGAT | **score=7.63** |
| *end-1* HMG | : | TCTTTGAA | **score=6.69** |
| POPHHOP HMG | : | CCTTTGAT | **score=8.36** |

Score matrix of Helper sites:

| A | -2.08 | -2.08 | -2.08 | 0.32 | 0.58 | 0.32 | 0.32 |
| --- | --- | --- | --- | --- | --- | --- | --- |
| C | -2.08 | 1.50 | 1.50 | -2.08 | 0.69 | -0.29 | -0.29 |
| G | 1.50 | -2.08 | -2.08 | 0.97 | -2.08 | 0.32 | -0.29 |
| T | -2.08 | -2.08 | -2.08 | -2.08 | -2.08 | -0.61 | -0.04 |

Alignment matrix of Helper sites:

| *ceh-22* Helper1 | : | GCCGAAA | **score=6.69** |
| --- | --- | --- | --- |
| *ceh-22* Helper2 | : | GCCGCTT | **score=5.51** |
| *psa-3* Helper | : | GCCGACA | **score=6.08** |
| *end-1* Helper1 | : | GCCAAAA | **score=6.04** |
| *end-1* Helper2 | : | GCCAAGT | **score=5.68** |
| *end-1* Helper3 | : | GCCACAC | **score=5.54** |
| POPHHOP Helper | : | GCCGCGG | **score=6.19** |
