# Supplementary material for: Distinct DNA Binding Sites Contribute to the TCF Transcriptional Switch in C. elegans and Drosophila
Source: PLoS Genet. 2014 Feb 6;10(2):e1004133. doi: 10.1371/journal.pgen.1004133 (PMC3916239; doi:10.1371/journal.pgen.1004133)
Supplement: Table S3 — List of 19 hits from a genome-wide search for HMG-Helper site clusters. Hits contained at least two Helper and one HMG site within 50 bp. The cutoffs for Helper and HMG sites were 5.51 and 6.69, using the weighed matrices shown in Table S2. The sequences shown could be in either forward or reverse orientation. The position corresponds to the 500 bp upstream of each gene that was searched; position 1 corresponds to −500 (from the first codon) onward toward position 500 (−1 from first codon). (DOCX) [file pgen.1004133.s010.docx]

| Gene name | Synomyn | Motif/Seq/Position | Motif/Seq/Position | Motif/Seq/Position | Motif/Seq/Position |
| --- | --- | --- | --- | --- | --- |
| 176230 | aak-1 | Helper/gggcggc/351 | Helper/cggcggc/354 | HMG/ttcaaaag/372 | Helper/gttcggc/402 |
| 176678 |  | Helper/gccgcgc/231 | HMG/atcaaaga/257 | Helper/gccaaaa/278 |  |
| 185390 |  | Helper/aagcggc/413 | HMG/ttcaaag/435 | Helper/gccgaag/446 |  |
| 186333 |  | Helper/tcttggc/69 | HMG/atccaaaga/100 | Helper/tggtggc/113 |  |
| 186716 |  | Helper/gccacaa/413 | HMG/cctttgaa/426 | Helper/gccacgg/462 |  |
| 3565758 |  | Helper/gccaaat/118 | HMG/ttccaaagc/135 | Helper/gccaaaa/149 |  |
| 173477 | fbxc-40 | Helper/gctcggc/195 | Helper/gccgcac/215 | HMG/gctttgaa/228 |  |
| 259701 |  | Helper/tatcggc/387 | HMG/ttcaaaag/412 | Helper/atgcggc/421 |  |
| 176588 |  | Helper/gccgcgg/143 | HMG/ttcaaagc/160 | Helper/gctcggc/186 |  |
| 3896816 |  | Helper/gccaaga/100 | HMG/ttcaaaga/115 | Helper/gccgcgc/150 |  |
| 173619 | fbxb-107 | Helper/gccgagc/363 | HMG/ttcaaaag/379 | Helper/gccgcac/389 |  |
| 186888 |  | Helper/gctcggc/180 | Helper/gccgcac/200 | HMG/gctttgaa/213 | Helper/tctcggc/228 |
| 176979 | znf9 | Helper/acgcggc/199 | Helper/gccacat/240 | HMG/tcttgat/249 | Helper/gccgact/270 |
| 187622 |  | Helper/gccacaa/140 | HMG/ttcaaagg/161 | Helper/gcgcggc/180 |  |
| 188003 |  | Helper/gccacgt/70 | HMG/cttttgaa/108 | Helper/gccacgc/117 |  |
| 188733 | sptf-2 | Helper/gccaaga/213 | HMG/atcaaaag/230 | Helper/gccgccg/254 |  |
| 13191444 |  | Helper/gccaaga/175 | HMG/ttcaaagg/183 | Helper/gccgaca/220 |  |
| 190533 | grd-16 | Helper/tggtggc/306 | HMG/atcaaaag/321 | Helper/gccacca/333 |  |
| 190541 | sprr-3 | Helper/gccaaaa/199 | HMG/atcaaaga/209 | Helper/gccgccg/220 | Helper/gccgaca/223 |
